# Molecular Structure of Cu(II)-Bound Amyloid-β Monomer Implicated in Inhibition of Peptide Self-Assembly in Alzheimer’s Disease

**DOI:** 10.1021/jacsau.2c00438

**Published:** 2022-11-11

**Authors:** Axel Abelein, Simone Ciofi-Baffoni, Cecilia Mörman, Rakesh Kumar, Andrea Giachetti, Mario Piccioli, Henrik Biverstål

**Affiliations:** † Department of Biosciences and Nutrition, Karolinska Institutet, Huddinge141 83, Sweden; ‡ Magnetic Resonance Center and Department of Chemistry, University of Florence, Via Luigi Sacconi 6, Sesto Fiorentino50019 , Florence, Italy; § Department of Biochemistry and Biophysics, The Arrhenius Laboratories, Stockholm University, Stockholm106 91, Sweden; ∥ Department of Physical Organic Chemistry, Latvian Institute of Organic Synthesis, RigaLV-1006, Latvia

**Keywords:** amyloid-β peptide, Alzheimer’s disease, copper ion, paramagnetic NMR, aggregation kinetics

## Abstract

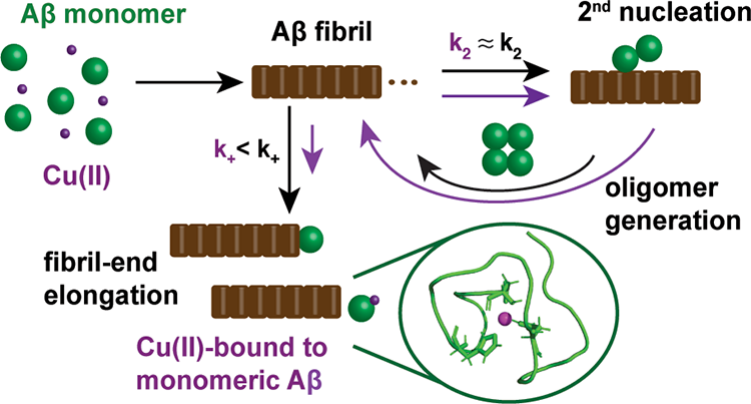

Metal ions, such as copper and zinc ions, have been shown to strongly modulate the self-assembly of the amyloid-β (Aβ) peptide into insoluble fibrils, and elevated concentrations of metal ions have been found in amyloid plaques of Alzheimer’s patients. Among the physiological transition metal ions, Cu(II) ions play an outstanding role since they can trigger production of neurotoxic reactive oxygen species. In contrast, structural insights into Cu(II) coordination of Aβ have been challenging due to the paramagnetic nature of Cu(II). Here, we employed specifically tailored paramagnetic NMR experiments to determine NMR structures of Cu(II) bound to monomeric Aβ. We found that monomeric Aβ binds Cu(II) in the N-terminus and combined with molecular dynamics simulations, we could identify two prevalent coordination modes of Cu(II). For these, we report here the NMR structures of the Cu(II)–bound Aβ complex, exhibiting heavy backbone RMSD values of 1.9 and 2.1 Å, respectively. Further, applying aggregation kinetics assays, we identified the specific effect of Cu(II) binding on the Aβ nucleation process. Our results show that Cu(II) efficiently retards Aβ fibrillization by predominately reducing the rate of fibril-end elongation at substoichiometric ratios. A detailed kinetic analysis suggests that this specific effect results in enhanced Aβ oligomer generation promoted by Cu(II). These results can quantitatively be understood by Cu(II) interaction with the Aβ monomer, forming an aggregation inert complex. In fact, this mechanism is strikingly similar to other transition metal ions, suggesting a common mechanism of action of retarding Aβ self-assembly, where the metal ion binding to monomeric Aβ is a key determinant.

## Introduction

Protein misfolding into fibrillar aggregates is a typical process found in the most common neurodegenerative diseases, such as the most prevalent cause of dementia, Alzheimer’s disease (AD).^1,2^ In AD, aggregation of the amyloid-β peptide (Aβ) leads to the formation of β-structured amyloid fibrils, which accumulate into amyloid plaques in the brains of AD patients.^2^ Environmental factors, such as pH, salt concentration, and cofactors, significantly influence the self-assembly process of Aβ and enhanced levels of metal ions were reported as co-deposits in amyloid plaques.^3−5^ Together with zinc and iron ions, copper ions are the most prevalent physiological transition metal ions. Due to their redox ability, they take on a key role as aggregation and toxicity modulating factor. Consequently, a dysregulated copper homeostasis has been associated with AD development,^6−9^ which is manifested by increased copper concentration in the amyloid plaques compared to the synaptic cleft and extracellular concentrations.^10^ Reduction of Cu(II) to Cu(I) can form reactive oxygen species (ROS) that may produce hydrogen peroxide, and that has been suggested as a neurotoxic process in AD.^6,11^ In addition, increasing evidence pinpoints the Aβ oligomers as the neurotoxic species.^1,12^ Cu(II) may stabilize oligomeric states^13,14^ and modulate their generation. Thus, unraveling the mechanistic details of Cu(II) interaction with Aβ is of great value to facilitate the understanding of neurotoxic processes in AD and enable the design of specific target and prevention strategies against the neurotoxic species, rather than overall Cu(II) chelation approaches.

Aβ is found in two most common forms with peptide lengths of 40 and 42 residues, referred to as Aβ40 and Aβ42, respectively. For both peptide isoforms, the underlying aggregation mechanism is dominated by secondary nucleation events, in addition to fibril-end elongation.^15,16^ Remarkably, specific inhibition of secondary nucleation events has been linked to attenuated toxic effects and this knowledge may hence enable specific intervention strategies.^17−20^

Typically, transition metal ions modulate the Aβ aggregation behavior in a concentration-dependent manner. In general, high concentrations of Zn(II) and Cu(II) have been associated with rapid formation of amorphous aggregates.^21−24^ In contrast, low concentrations of these metal ions lead to retarded formation of Aβ fibrils.^24−28^ Our previous studies showed that the mono- and divalent transition metal ions Ag(I) and Zn(II) mainly decrease the Aβ aggregation rate by rather specifically inhibiting fibril-end elongation,^25,29^ arising the question whether also Cu(II) ions feature a similar mechanism of action.

The hydrophilic N-terminus of the Aβ peptide is the preferred binding site of the most common metal ions, where three histidines are involved as binding ligands.^11,30,31^ For Zn(II) ions, the fourth binding ligand has been assigned to the N-terminal aspartic acid, where E11 may be an alternative binding ligand.^30−33^ A very similar metal coordination is present for Ag(I) ions.^29,34^ For the non-paramagnetic metal ions Zn(II) and Ag(I), a dynamic metal–ion bound complex with monomeric Aβ is formed, where exchange between a metal ion-bound state and the free state is observable by relaxation dispersion nuclear magnetic resonance (NMR).^25,29^

In the case of Cu(II), different binding modes have been suggested, involving for instance the terminal amine group, the backbone CO groups of D1 or A2, and the imidazole rings of histidines in different combinations.^35−39^ Non-paramagnetic Cu(I), which exhibits weaker binding to Aβ compared to Cu(II),^40^ was shown to be coordinated by the imidazole rings of two or three histidines.^34^ For Zn(II) ions, a solution NMR structure of the Zn(II)-Aβ(1–16) complex was published.^41^ In contrast, the paramagnetic nature of Cu(II) has made interaction studies using conventional NMR experiments difficult due to extreme line broadening of resonances in close proximity to the paramagnetic ion. Notably, two studies used NMR chemical shifts perturbations and 1D paramagnetic experiments to obtain some insights into the coordination mode of Cu(II),^42,43^ yet no molecular structure of the Cu(II)-bound Aβ complex is deposited.

In this study, we report an NMR structure representing a molecular model of Cu(II) bound to the first 23 N-terminal residues of full-length Aβ40, exclusively based on paramagnetic structural constraints. Notably, while most previous Cu(II) coordination studies have used shorter, N-terminal Aβ sequences,^35−38^ we investigated here full-length Aβ40. In addition to structural studies, we conducted a global fit analysis of Aβ40 and Aβ42 aggregation kinetics to elucidate which nucleation steps are modulated by Cu(II). These results were rationalized in a model unraveling the molecular mechanism of Cu(II) modulation of Aβ self-assembly. Together with previous results for Zn(II) and Ag(I),^25,29^ these findings suggest a common mechanism of action of inhibiting Aβ fibril formation by coordination of transition metal ions to the Aβ monomer.

## Results and Discussion

### Monomeric Aβ Binds Cu(II) in the N-Terminus

**Figure 1:**
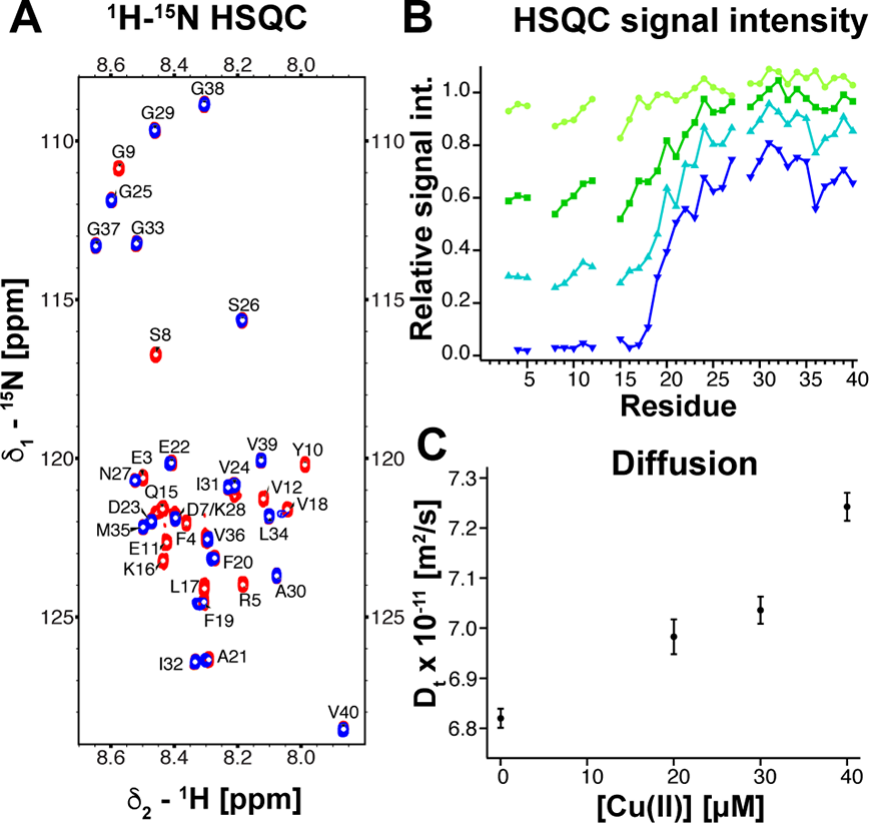
Cu(II) binds to the N-terminus of Aβ40 and forms a compact complex. (A) ^1^H-^15^N HSQC spectra of 75 μM Aβ40 in 10 mM HEPES, pH 7.2, with (blue) and without (red) 100 μM Cu(II) at 281 K. (B) Concentration-dependent attenuation of N-terminal signals upon titration of 40 (light green), 60 (green), 75 (cyan), and 100 μM (blue) Cu(II) concentrations. (C) The translational diffusion coefficient of Aβ40 increases with increasing Cu(II) concentration, indicating a smaller hydrodynamic radius of the Cu(II)-bound state.

We first study the binding of Cu(II) to monomeric Aβ40 using ^1^H-^15^N HSQC NMR titration experiments. The experiments were performed with 75 μM Aβ40 in 10 mM HEPES buffer, pH 7.2, at 281 K and revealed a Cu(II) concentration-dependent loss of the NMR signals of the N-terminal residues (Figure 1A,B). At 100 μM Cu(II), the ^1^H-^15^N HSQC signal intensity plotted against the peptide sequence decreases steeply between residues 18 to 24 and an almost complete loss of cross-peak intensities is present for all residues 1 to 17, in good agreement with a previous report.^44^ Interestingly, we observe small chemical shift changes for the residues 18 to 21 (Figure 1A and Figure S1A). These changes suggest that these residues undergo chemical exchange in the slow exchange regime between a metal ion-bound and a free state. Also ^1^H-^13^C HSQC experiments revealed a specific signal loss of N-terminal residues also including D1, whose signal was not visible in the ^1^H-^15^N HSQC spectrum (Figure S1B).

To investigate further the induced structural change upon Cu(II) binding, we conducted pulse field gradient diffusion experiments^45,46^ at different Cu(II) concentrations. Here, we observed a concentration-dependent increase of the translational diffusion coefficient from (6.82 ± 0.02) to (7.24 ± 0.03) × 10^–11^ m^2^/s for 0 to 40 μM Cu(II) concentration (Figure 1C). This corresponds to a decrease of the apparent hydrodynamic radius from 16.8 to 15.8 Å, suggesting a more compact structure upon Cu(II) binding.

The origin of the strong attenuation of N-terminal signals might be attributed to line broadening caused by chemical exchange processes and/or the paramagnetic nature of Cu(II). Interestingly, we have previously reported similar disappearance of N-terminal resonances for non-paramagnetic metal ions, such as Zn(II) and Ag(I), where we attributed the signal attenuation to a dynamic exchange process.^25,29^ Yet, the signal loss was less uniform for these metal ions and more pronounced for residues close to the binding ligands. In contrast, in the case of Cu(II), we observed a much stronger and uniform line broadening for the residues 1 to 17. Hence, the paramagnetic properties of Cu(II) is expected to be the primary cause of the extreme line broadening.

To investigate whether we can detect any contribution of line broadening due to chemical exchange, we recorded ^15^N Carr–Purcell–Meiboom–Gill (CPMG) relaxation dispersion measurements. These experiments typically detect exchange in the micro- to milli-second time scale. We recorded ^15^N-CPMG relaxation dispersion profiles at low Cu(II) concentrations, where around 40% of the initial N-terminal signal intensity is lost, using 11 different CPMG frequencies (Figure S2). Under these conditions, we observed flat profiles, suggesting that no exchange is detectable for this Cu(II) concentration and time scale by CPMG relaxation dispersion experiments. In comparison, both Zn(II) and Ag(I) ions exhibit high amplitude relaxation dispersion profiles for similar reduction of signal intensity.^25,29^ Hence, we conclude that the line broadening in the presence of Cu(II) indeed primarily originates from the paramagnetic relaxation contribution.

Based on the paramagnetic relaxation property of Cu(II), we subsequently employed specific NMR experiments that are tuned to characterize systems containing paramagnetic metal ions.

### Elucidating Cu(II) Binding by Paramagnetic Relaxation Enhancement NMR Experiments

Paramagnetic relaxation enhancement (PRE) provides structural insights into metalloproteins that contain a paramagnetic metal ion.^47−50^ Several factors may contribute to paramagnetic relaxation; for a non-blue Cu(II) chromophore like the present case, the observable PRE is fully due to magnetic dipolar interactions between the nucleus and the paramagnetic metal ion. This allows to correlate the measured PRE value with *r^–6^*, where *r* is the distance between the nucleus and the paramagnetic center.^47−50^

**Figure 2:**
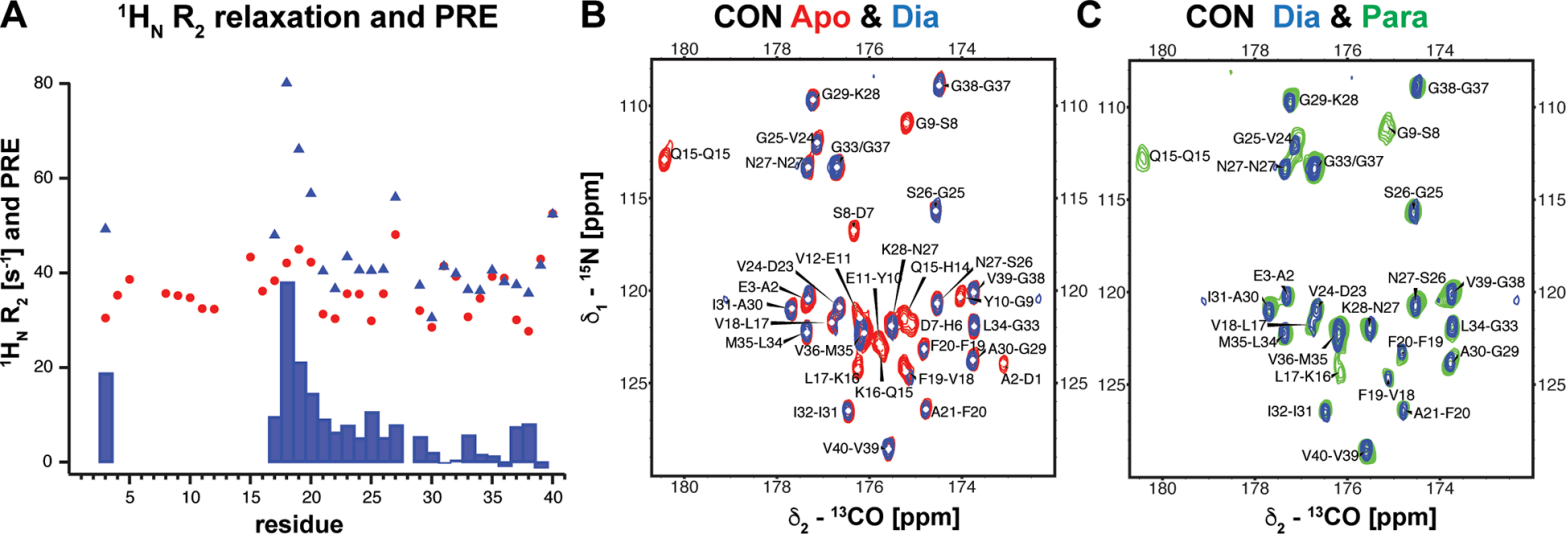
Paramagnetic NMR experiments decrease the blind sphere around the paramagnetic center. (A) ^1^H_N_-R_2_ rates of Aβ40 with (blue triangles) and without (red dots) 100 μM Cu(II) and PRE corresponding to the difference of these two rates (shown as bars). (B) CON experiment of Aβ with (blue, dia-state) and without (red, apo-state) 100 μM Cu(II). (C) CON optimized for paramagnetic agents (green, para-state) exhibits the recovery of three cross-peaks that are lost in the diamagnetic version (blue).

Here, we measured the longitudinal and transverse relaxation rates, ^1^H_N_-R_1_ and ^1^H_N_-R_2_, respectively, of the amide protons of 75 μM Aβ40 without and with 100 μM Cu(II) present. Due to the loss of signals of the N-terminal residues at 100 μM Cu(II) concentration, we could only measure ^1^H_N_-R_1_ relaxation rates from residue 17 (Figure S3). We found that the ^1^H_N_-R_1_ rates of those residues are very similar without (referred to as apo-state) and with 100 μM Cu(II) (referred to as para-state). Compared to the longitudinal PRE, the transverse PRE has been assigned as the more reliable and useful way to extract structural information.^47^ Indeed, when recording transverse PRE based on the ^1^H_N_-R_2_ relaxation rates of the apo- and Cu(II) para-states (Figure 2A and Figure S4), we could detect PRE values for the residues E3 and L17-A21 that are among those close to the paramagnetic center (Figure 2A). The PRE values can be translated to distance constraints using the dependence of the R_2_ rate on the spectral density function (see the Methods Section).^49,51^ The obtained PRE distances are in the range of 7.6 to 9.1 Å (Supporting Information,Table S1) and give valuable distance constraints for subsequent structure determination.

### Decreasing the Blind Sphere around the Paramagnetic Cu(II) Ion by Tailored NMR Experiments

While NMR signals from residues close to paramagnetic centers are lost in conventional NMR experiments, modified pulse sequences of 2D ^1^H-^15^N HSQC experiments^52^ and other double and triple resonance NMR experiments^53^ can be used to obtain information about residues in short distance to the paramagnetic metal ion. Compared to a conventional ^1^H-^15^N HSQC experiment, the modified ^1^H-detected para-^15^N HSQC starts with an inversion recovery filter before the INEPT step, and the INEPT transfer period is drastically shorten to account for the fast relaxation times.^52^ Further, the signals are directly recorded after the INEPT step, without refocusing the signals, as anti-phase components, and processed in dispersion mode.^52^ Similarly, also ^13^C-detected 3D NMR experiments para-CON, para-CaCO, and para-CbCaCO can be optimized for very fast relaxation resonances,^53^ enabling the detection of nuclei close to the paramagnetic center. Thus, by applying these para-experiments, the blind sphere around the paramagnetic ion can be substantially reduced. Moreover, structural constraints can be deducted from resonances that are present in conventional, diamagnetic experiments (referred to as dia-experiments), related to nuclei outside the blind sphere of dia-experiments, and for those nuclei that appear only in para-experiments, which can be assigned to atoms between the blind spheres of para- and dia-experiments.

Here, we applied ^1^H-detected para-^15^N HSQC and ^13^C-detected para-CON, para-CaCO, and para-CbCaCO experiments, in comparison to dia-experiments, to characterize the Cu(II)-bound state of monomeric Aβ40. As expected, we could observe resonances of fast relaxing paramagnetic signals in addition to strong signals present in dia-experiments resulting from highly flexible residues (Figure S5). Remarkably, the signals of amide protons of E3, F4, G9, K16, and F19, as well as the side chain ^1^H_N_ protons of Q15, could be recovered in ^1^H-detected para-^15^N HSQC compared to the dia-spectrum.

This gives useful distance constraints since, on the one hand, these nuclei are close enough to the paramagnetic center to be broadened beyond detection in dia-experiments and, on the other hand, are far enough from the paramagnetic center to be recovered in para-experiments. Hence, these nuclei can be constrained by lower and upper distance limits of 6.5 to 9.0 Å (Table S2). Similar information can be obtained from the analysis of dia *vs* para ^13^C-detected experiments. In the para-CON spectrum, the intensity of three resonances (S8, K16, & L17) could be significantly increased compared to the dia-spectrum (Figure 2B) and therefore constrained to a 4.5 to 9 Å shell around the metal ion. Applying the para-CaCO experiment recovered three additional resonances, the C^α^ of S8 and Y10 and the C^γ^ of Q15, compared to the dia-spectrum (Figure S6). In the para-CbCaCO experiment, five resonances could be recovered compared to the dia-experiment (Figure S7).

In addition to these upper and lower distance constraints, also signals that cannot be recovered in the para- compared to the dia-spectrum give useful distance constraints, since these nuclei are in very close proximity to the metal ion. In the para-CON spectrum, we found that the carbonyl carbons of D1, D7, Y10, E11, and Q15 are not visible and, hence, we constrained these nuclei to an upper distance limit of 6.5 Å to the paramagnetic center (Figure 2 and Table S2). Likewise, five resonances were not recovered in the para-CaCO and used as distance constraints applying the same distance constraints as for the CON experiments (Figure S6 and Table S2). In the para-CbCaCO experiment, 15 signals were still invisible, associated to nuclei close to the Cu(II) ion (Figure S7 and Table S2).

In summary, by performing ^1^H- and ^13^C-detected para-experiments, distance constraints could be applied for 42 nuclei, which were used in subsequent structural calculations.

### Molecular Structure of the Aβ’s N-Terminus Folded around the Cu(II) Ion

**Figure 3:**
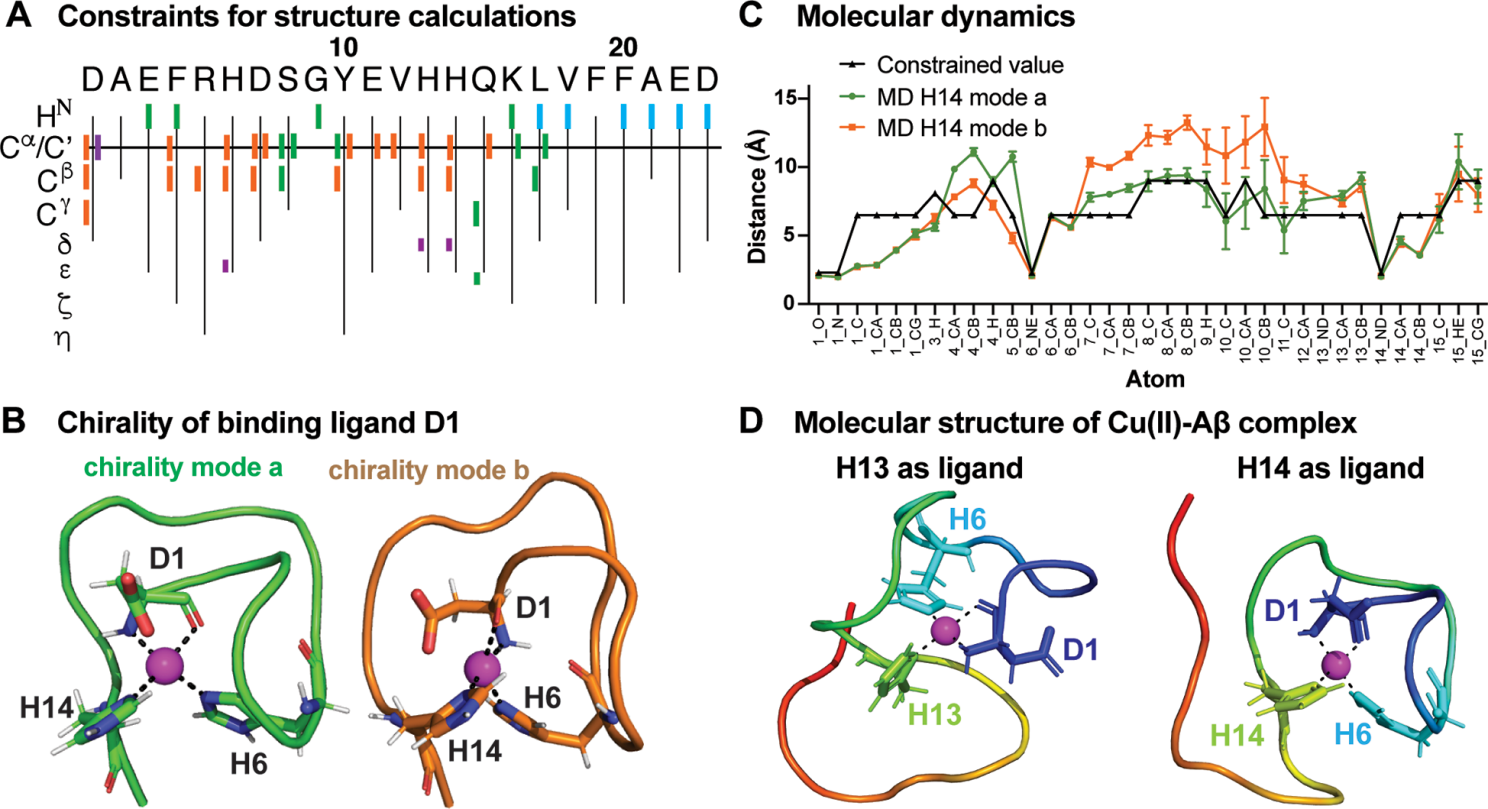
NMR structures and MD simulations of the Cu(II)-bound Aβ complex. (A) Overview about all constraints used for structure calculations. Binding ligands are colored in magenta, nuclei exhibiting PRE in cyan, nuclei within a 4.5 to 9.0 Å (for ^13^C atoms) or 6.5 to 9.0 Å (for H^N^-atoms) shell in green, and nuclei within the blind sphere of NMR para-experiments (<6.5 Å) in orange. (B) Binding ligands of Cu(II) visualizing the two different chirality modes of the binding ligand D1 here shown for H14 as the fourth ligand. Both chirality modes also exist for the alternative coordination with H13 as the fourth ligand. (C) MD simulations for the chirality modes *a* and *b* compared to the experimental constraints for residues 1 to 15 for the coordination with H14 as fourth ligand (for H13 see Figure S8), suggesting chirality mode *a* as the preferred coordination. (D) High-resolution structures of the first 23 N-terminal residues of Aβ40 encapsulating the Cu(II) ion for the coordinations of chirality mode *a* with H13 and H14 as fourth ligand, respectively.

To determine a molecular structure of Aβ in complex with Cu(II), we applied lower and upper distance constraints, obtained from PRE experiments and ^1^H- and ^13^C-detected para-NMR experiments, using the CYANA software.^54^ All distance constraints are summarized in Figure 3A and Table S2. Due to the lack of constraints for the middle and C-terminal part of the peptide sequence, we limited the structural calculation to the first 23 N-terminal residues of Aβ.

With the final goal to obtain a high-resolution structural model of Aβ bound to Cu(II), we had to identify the potential binding ligands, which need to be constrained to close proximity to the metal ion. We could exclude potential binding ligands whose resonance are either directly visible or whose neighboring resonances are observable in our experiments. Consequently, based on our recorded spectra, we excluded A2, E3, D7, Y10, and E11 as potential ligands since their resonances were either directly observed by paramagnetic experiments (for E3 and Y10) or direct neighboring residues were visible (for A2, D7, and E11). Resonances of these potential binding ligands or of neighboring residues are listed in Table 1. On the contrary, we observed a signal loss of C^α^ and C^β^ in CbCaCO and CaCO experiments of D1, H6, H13, and H14 and of the carbonyl carbon of D1 in CON. Importantly, these signals could not be recovered using para-NMR experiments since the corresponding resonances remain in the blind sphere, suggesting that these residues are in very close proximity to the paramagnetic metal ion. Hence, based on these findings, we assigned the nitrogen atoms in the imidazole ring of the three histidines as well as D1 as Cu(II) binding ligands.

**Table 1:** Alternative Binding Ligands. The Alternative Binding Ligands E3, D7, Y10 and E11 Are either Directly Visible in Paramagnetic Experiments or Direct Neighboring Residues Are Observable in Paramagnetic Experiments

Alternative ligands	Residue visible in	Visible neighboring residues	Shortest distance to assigned ligands
A2	*Not visible*	E3 in ^1^H_N_-PRE	1 aa to D1
		E3 in para ^1^H-^15^N HSQC	
E3	^1^H-PRE	*Directly observable*	2 aa to D1 and H6
	paraHSQC		
D7	*Not visible*	S8 in paraCaCO	1 aa to H6
		S8 in paraCaCbCO	
Y10	paraCaCO	*Directly observable*	3 aa to H13
	paraCbCaCO		4 aa to H6 and H14
E11	*Not visible*	Y10 in paraCaCO	2 aa to H13
		Y10 in paraCbCaCO	3 aa to H14

This result is in good agreement with previous, mainly electron paramagnetic resonance (EPR), studies suggesting that at pH 7.2, as used in our studies, one coordination mode largely prevails (component I), while another coordination mode (component II), which involves the CO group of A2, is more favored at higher pH values.^55,56^ In the literature, component I converges to a 3N1O equatorial coordination made of the NH_2_ terminus, the backbone CO group from D1, the imidazole ring of H6, and that of H13 or H14 (reviewed in ref (55)). EPR studies indicated that these two coordination modes equilibrate in solution as predominant species with respect to the species involving the simultaneous presence of H13 and H14 as Cu(II) ligands,^35,38^ which was supported by measuring the binding affinities of histidine modified Aβ variants.^57^ Hence, we neglected this coordination in our structural calculations. Noticeably, which of the nitrogens in the imidazole ring of the histidines is the binding nucleus is not determinable from our current NMR data. However, FTIR-based data support that N_ε_ of H6 and N_δ_ of H13 or H14 as binding nuclei.^58^ In conclusion, we assigned as binding ligands the nitrogen of the NH_2_ terminus, the amide oxygen of D1, N_ε_ of H6, and N_δ_ of H13 or H14 and included these structural constraints in two CYANA structure calculations with H13 or H14 as fourth ligand, where we restricted the distance of the binding nuclei to an interval of 1.8 to 2.3 Å.

The obtained best structural conformer of the two structure calculations was then used as a starting point for molecular dynamics (MD) simulations to properly define the coordination geometry of the Cu(II) ion.

MD simulations were thus performed on the two binding modes with H13 or H14 as the fourth binding ligand, respectively. In the MD simulations, we also took into account that the formation of the metal complex by Cu(II) coordination to the NH_2_ terminus and the backbone CO group from D1 generates two possible chiral conformers. Thus, four MD simulations were performed with H13 or H14 in the two possible chirality modes of the metal complex formed by D1 (named mode *a* and *b*, Figure 3B). While MD simulations were shown to highly depend on the applied force field,^59^ we used here a previously applied approach for the Cu(II)–Aβ complex^60^ to reduce the error source in usage of computational methods. We found that all calculated structures remain overall stable over a time frame of 1 ms (Figure S8), and that one of the two chirality modes of the metal complex formed by D1, *i.e.,* mode *a*, is in much better agreement with the paramagnetic distance constraints. Indeed, several and large violations are present for the structural part between the binding ligands H6 and H14 in the chirality mode *b* when H14 is the fourth ligand (Figure 3C). This effect was not observed for the chirality mode *a*, which fulfilled paramagnetic distance constraints in both H13 and H14 coordinations for the structural part comprising residues 1–15 (Figure 3C and Figure S8).

Applying the chirality mode *a*, the five best structural conformers obtained by the two CYANA calculations with H13 and H14 as a ligand were then restrained energy-minimized in water by AMBER. For the core structures (residue 1 to 14), we obtained an average backbone RMSD to mean of 1.92 and 2.13 Å for H13 and H14, respectively (see Table S3 for all structure parameters). The best conformers of the AMBER energy-refined structures are shown in Figure 3D, and the structural ensembles are visualized in Figure S9. The atomic coordinates have been deposited in the Protein Data bank with PDB ID codes 8B9Q and 8B9R for H13 and H14 as fourth ligand, respectively.

### Cu(II) Inhibits Aβ Aggregation in a Concentration-Dependent Manner

**Figure 4:**
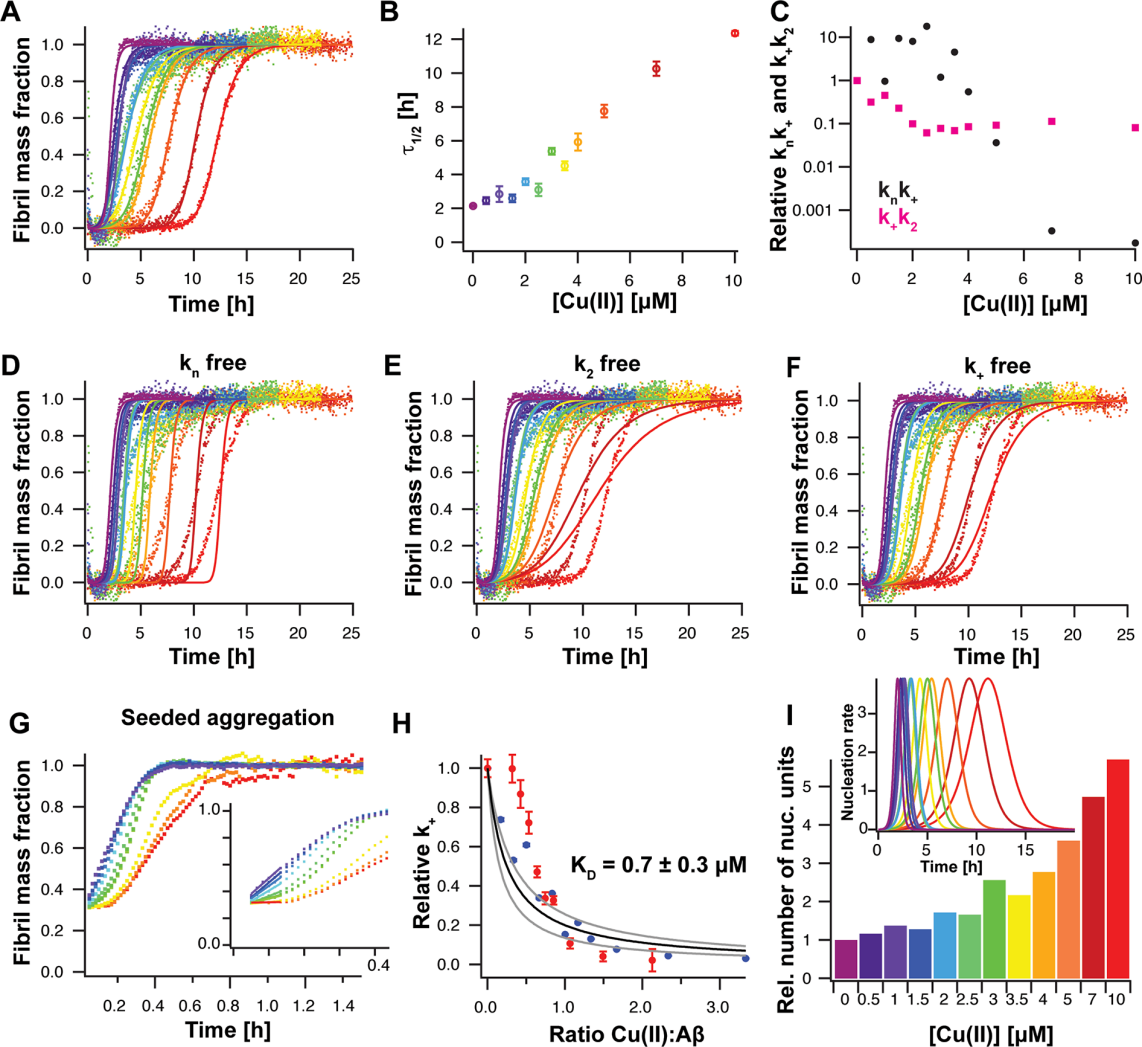
Cu(II) inhibits Aβ42 aggregation by specifically retarding fibril-end elongation. (A) Aggregation kinetics of 3 μM Aβ42 in 10 mM sodium-phosphate buffer, pH 8, during quiescent conditions in the presence of different Cu(II) concentrations from 0 (violet) to 10 μM Cu(II) (red). The aggregation traces were individually fitted with a nucleation model, including primary and secondary nucleation as well as fibril-end elongation. (B) Aggregation half times, τ_1/2_, obtained from sigmoidal fits of the aggregation traces in panel (A). (C) Relative combined rate constant *k_n_k*_+_ (black) and *k*_+_*k*_2_ (pink), related to primary and secondary nucleation processes, respectively, as obtained from fits shown in panel (A). While the parameter *k*_+_*k*_2_ only shows a small variation with Cu(II) concentration, the parameter *k_n_k*_+_ changes over several orders of magnitude, indicating that Cu(II) primarily affects primary nucleation and/or fibril elongation. (D–F) Global fit analysis of aggregation traces where the fit parameters were constrained such that only one nucleation rate constant is the sole fitting parameter, *i.e*., *k_n_* in panel (D), *k*_2_ in panel (E), and *k*_+_ in panel (F), revealing the best fit for *k*_+_. (G) Highly seeded aggregation kinetics of 3.2 μM Aβ42 in the presence of 1.5 μM pre-formed fibrils. Under these conditions, the initial slope (inserted graph as zoom of the first 0.4 h) of the aggregation traces is proportional to *k*_+_. (H) Relative elongation rate as obtained from seeded aggregation kinetics (panel G, red) and from global fit analysis (panel F, blue) as a function of the Cu(II):Aβ ratio. The data could be fitted to a model describing monomer attachment to the fibril ends, revealing an apparent dissociation constant of *K_D_*= 0.7 ± 0.3 μM, where the gray curves represent the error range, which gives the error of *K_D_*. (I) Relative number of nucleation units at different Cu(II) concentrations obtained from the integral of the nucleation rate (inserted graph). The nucleation rate is calculated from the parameters of the kinetic analysis and given in units of M s^–**1**^ ×10^–14^.

We further seek to shed light on how the Cu(II)-bound Aβ complex modulates the aggregation mechanism of Aβ. The kinetics of protein self-assembly can be monitored using the amyloid-binding fluorescent dye thioflavin T (ThT). Both Aβ isoforms, Aβ40 and Aβ42, typically exhibit a sigmoidal aggregation behavior,^15,16^ which is characterized by the aggregation half time **τ**_1/2_. Therefore, we first chose Aβ42 for the aggregation studies (Figure 4).

**Figure 5:**
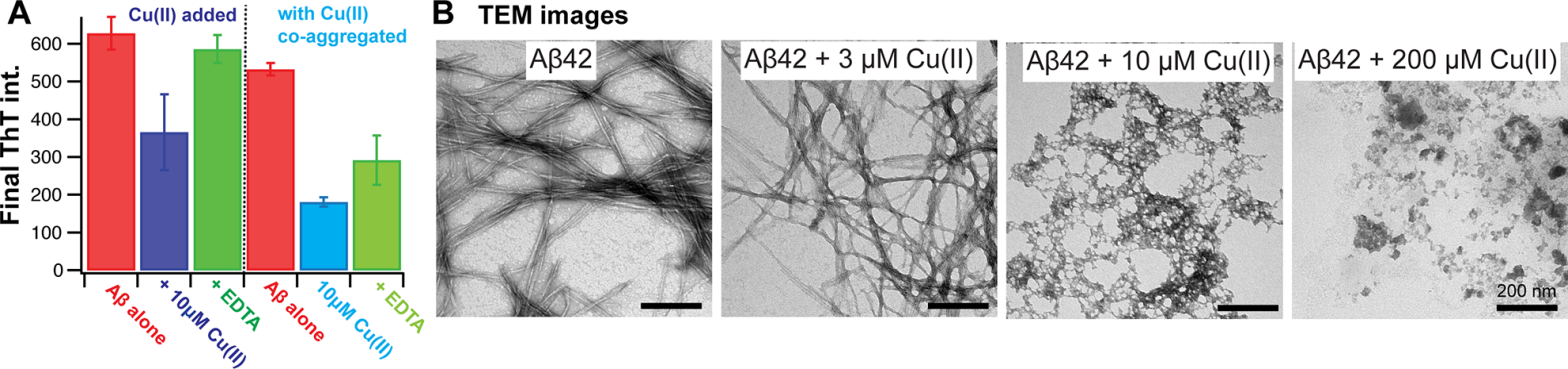
Final ThT intensity and TEM images of Cu(II)-Aβ42 aggregates. (A) Final ThT intensity of fibrils without Cu(II) (red) and upon addition of 10 μM Cu(II) (blue) and 5 mM EDTA (green) (left panel) or co-aggregated in the presence of 10 μM Cu(II) (cyan, right panel) and subsequent addition of 10 mM EDTA (light green, right panel), showing recovery of signal intensity upon EDTA addition. (B) TEM images were recorded without further storage at the end-points of the aggregation kinetics experiments, revealing clear fibril morphologies without and with low Cu(II) concentrations. In contrast, at 200 μM Cu(II) mostly amorphous structures are visible, which only exhibit very low ThT fluorescence.

We recorded the aggregation traces of 3 μM Aβ42 in 20 mM sodium-phosphate buffer, pH 8, in the presence of different Cu(II) concentrations from 0 to 10 μM (Figure 4A and Figure S10) and found that Cu(II) inhibits Aβ42 aggregation in a concentration-dependent manner, where the aggregation half-time increases with increasing Cu(II) concentration (Figure 4B) and that the final ThT fluorescence intensity is modulated by Cu(II). The decreased ThT fluorescence might either be caused by formation of another fibril morphology, which is less ThT active, or interaction of Cu(II) with ThT causing fluorescence quenching. Notably, Cu(II) has been reported to quench the fluorescence of ThT in the literature,^23,26^ resulting in an attenuated final ThT amplitude dependent on Cu(II) concentration. To confirm the quenching effect, we added different concentrations of Cu(II) to mature Aβ42 fibrils and found that the final intensity is decreased in a Cu(II) concentration-dependent manner, e.g., around 60% for 10 μM Cu(II) (Figure 5A and Figure S11A). Remarkably, subsequent addition of 5 mM EDTA basically completely recovers the original end-point fluorescence of Aβ42 fibrils without Cu(II) (Figure 5A). Since no change of the Aβ42 fibrils morphology is expected, the modulation of the ThT final intensity can be attributed to a quenching effect. Also, fibrils co-incubated with Cu(II) exhibited a decreased final ThT amplitude (Figure 5A and S11B), where addition of 10 mM EDTA to the final states of 5 and 10 μM Cu(II) co-aggregated samples increased the final ThT intensity, yet remain significantly below the value of mature Aβ42 fibrils. This indicates that a smaller proportion of Cu(II) was still bound and not accessible to EDTA binding and/or the fibril morphology has changed resulting in less ThT binding. Together, these results suggest that the attenuated final ThT signal intensity for addition of Cu(II) to mature fibrils is caused by Cu(II) quenching, while the modulated ThT amplitude for Cu(II) co-incubated fibrils might be caused by a modulated fibril morphology in addition to the ThT quenching effect. Importantly, also other factors such as clustering of amyloid fibrils, light scattering, and fibril sedimentation influence the recorded final ThT signal, making the final ThT intensity a less reliable parameter compared to the aggregation half time.

We repeated the kinetics experiments with the shorter Aβ40, which aggregates slower than Aβ42 (Figures S12 and S13). Similarly as for Aβ42, Cu(II) prolonged the aggregation half-time in a concentration-dependent manner (Figure S12B).

To obtain further insights on the inhibition mechanism of Cu(II) on Aβ aggregation, we analyzed the recorded aggregation traces in more detail.

### Fibril-End Elongation Is Predominantly Retarded by Cu(II)

In general, protein aggregation is governed by a nucleation mechanism including different nucleation events.^61,62^ Formation of small nucleation units by monomers is referred to as primary nucleation, which is described by the nucleation rate constant *k_n_*. Further, the fibril surface may act as catalytic site that promotes generation of new nucleation units, described as secondary nucleation with the rate constant *k_2_*. In addition, the rate of fibril growth at the fibril-ends is determined by the elongation rate constant *k_+_*.

For Aβ40 and Aβ42, the dominating aggregation mechanism was assigned to secondary nucleation using a global fit analysis with a nucleation model.^15,16^ Here, we applied this kinetics analysis and individually fitted the aggregation traces using a model, where the two combined rate constants  and  are the free fitting parameters. The fits exhibit a strong dependence of the relative fitting parameter *k_n_k*_+_ on the Cu(II) concentration varying several orders of magnitude, while the relative fitting parameter *k*_+_*k*_2_ is only slightly affected (Figure 4C and Figure S12). This analysis suggests that either nucleation steps associated to primary nucleation and/or fibril-end elongation are modulated by the presence of Cu(II).

To test the contribution of each individual rate constant, we fitted the aggregation traces globally, where one fitting parameter is held to a constant value (which was determined by the aggregation trace without copper ions) and the other one is allowed to vary across different Cu(II) concentrations (Figure 4D–F and Figure S12D−F). This approach results in that one nucleation rate constant is the sole free fitting parameter.^25,63^ For Aβ42, this analysis revealed the best fit for *k_+_* as the free fitting parameter, reflected in the lowest χ^2^ value of 23.4 compared to 39.8 for *k_2_* and 50.2 for *k_n_*, suggesting that Cu(II) mainly reduces the rate of fibril elongation, in good agreement with another recently published study that indicated a major effect on Aβ42 fibril elongation by Cu(II)^26^. Also for Aβ40, a modulation of only the elongation rate constant *k_+_* described best the kinetic behavior (Figure S12D–F).

To further confirm that fibril-end elongation is affected by Cu(II), we used highly seeded aggregation kinetics experiments (Figure 4G and Figure S12G). Due to the large amount of fibrillar species, the major nucleation mechanism is predominately fibril elongation.^62^ Under these conditions, the aggregation trace exhibits a concave shape and the initial slope is proportional to the fibril-end elongation rate. We found that the relative elongation rate determined from these seeding experiments (Figure 4G and Figure S12G) exhibits a similar dependence on the Cu(II):Aβ ratio as the one determined from the global fit analysis (Figure 4H and Figure S12H). Hence, we concluded that it is indeed mainly fibril-end elongation that is retarded by the presence of Cu(II) for both Aβ40 and Aβ42.

The observed reduction in fibril-end elongation can be fitted to a model that describes the effect of metal-ion binding to Aβ monomers on the elongation rate,^64^ i.e., in this model, Cu(II) binding to monomeric Aβ peptides transiently removes them from the aggregation-prone peptide pool and this process affects monomer-dependent fibril-end elongation, described by an apparent dissociation constant *K*_*D*_^app^. When fitting the data with this model, we can estimate an apparent dissociation constant of Cu(II) binding to Aβ monomers, which is *K*_*D*_^app^ = 0.7 ± 0.3 μM for Aβ42 (Figure 4H) and *K*_*D*_^app^ of 1.4 ± 0.2 μM for Aβ40 (Figure S12H). Additionally, performing the aggregation kinetics with another amyloid-binding dye, pentameric formyl thiophene acetic acid (pFTAA),^65^ resulted in qualitatively similar results (Figure S12). Hence, the apparent *K*_*D*_^app^ is very similar for both isoforms obtained at pH 8 and 7.2, for Aβ42 and Aβ40, respectively.

In the literature, a broad range of apparent and conditional *K_D_* values has been reported for Cu(II) binding to diverse Aβ peptides spanning from 10^–10^ to 10^–6^ M (reviewed in ref (66−68)). A conditional *K_D_* value in the order of 10^–10^ M is in general accepted for monomeric Aβ,^66−68^ whereas apparent *K_D_* values highly dependent on experimental conditions (such as buffer contribution and concentration range) are often closer to 10^–6^ M.

Effects of unspecific and/or secondary binding contributing to the apparent *K_D_* shall not be excluded. The obtained apparent *K*_*D*_^app^ is within this range and in particular for full-length Aβ several studies have published lower binding affinities in the same order of magnitude as obtained here.^66−68^ Of note, the apparent dissociation constant described here refers to Cu(II) binding to the Aβ monomer and does not include binding to Aβ aggregates.

In summary, our detailed analysis of aggregation kinetics data of Aβ40 and Aβ42 reveals that predominantly one nucleation event – the fibril-end elongation – is affected by the Cu(II) ion.

### Kinetic Analysis Predicts Promotion of Aβ Oligomer Generation by Cu(II)

Production of oligomer Aβ species has been linked to cytotoxic effects,^1,12^ and metal ions have been shown to modulate oligomeric states of Aβ.^13,14^ Here, we applied a model to calculate the number of new nucleation units, which gives an estimate about the number of low-molecular weight oligomers generated during the aggregation kinetics.^17^ In this model, the reaction profile is determined by the specific nucleation rate constants *k_n_*, *k*_2_, and *k_+_* and can be calculated from the parameters of the kinetic analysis (Figure 4A–G), revealing a Cu(II)-dependent effect on the aggregation reaction (Figure 4I, inset). The specific inhibition of the fibril-end elongation rate by Cu(II) results in a delayed time when the maximum of the reaction profile is reached. More importantly, the number of new nucleation units is drastically increased, which is reflected by the increased integral of the nucleation rate over the reaction time. Indeed, the integral over the reaction time increases in a Cu(II) concentration-dependent manner, with a > 5 times increase at 10 μM Cu(II) for Aβ42 (Figure 4I). The corresponding calculations for Aβ40 (Figure S12I) also showed a ca. 3-fold increase of the number of nucleation units at 1:1 ratio, which is approximately the same as for Aβ42 (Figure 4I).

This analysis agrees well with the theoretical predictions performed for chaperone-mediated aggregation kinetics of Aβ.^17^ Interestingly, in several studies, the effect on oligomer generation obtained from kinetic analysis could be correlated to modulation of toxic effects by molecular chaperones^17,18,20^ and the effect of antibodies.^19^ Hence, Cu(II) should enhance toxic effects of Aβ-associated toxicity by elevated oligomer production. Indeed, Cu(II) has been reported to increase Aβ-induced cell toxicity by modulated oligomer formation.^13,14^ Yet, also formation of ROS species triggered by Cu(II) is linked to neurotoxicity (reviewed in^24,36^), suggesting the promoted oligomer generation as a potential additional factor underlying Cu(II)-Aβ associated toxic effects.

### TEM Analysis Revealed Fibril Structures at Low Cu(II) and Amorphous Aggregates at Higher Cu(II) Concentrations

We limited the aggregation kinetic analysis to low Cu(II) concentrations (≤10 μM), which showed a modulated ThT final intensity due to ThT quenching and/or change in fibril morphology. In contrast, at higher Cu(II) concentrations, the formation of amorphous aggregates has been reported.^24^ Hence, we next set out to study the final aggregate morphology at a broad Cu(II) concentration range using transmission electron microscopy (TEM).

We prepared TEM grids of Aβ aggregates at different Cu(II) concentrations obtained at the end stage of the aggregation kinetics (Figure 5). The images showed clear fibrillar morphology at 0 and 3 μM Cu(II). At 10 μM Cu(II), the aggregate structures exhibit short intertwined fibrils. At higher Cu(II) concentrations, *i.e.,* ≥30 μM Cu(II), only very small amounts of fibrils are visible in the TEM images, and instead amorphous aggregates are seemingly the major species.

In the literature, at molar ratios of Cu(II):Aβ ≫ 1, a second binding site has been proposed to become significantly populated,^24^ which promotes the formation of amorphous aggregates. Indeed, also our ^1^H-^15^N HSQC titration experiments with Aβ40 indicate a second weak binding site around residue D23 (Figure 1B), which might result from charge–charge interactions. Hence, the second binding site might facilitate intermolecular interactions via Cu(II)-mediated bridges.^23,69^

### Comparison to Other Metal Ions

**Figure 6:**
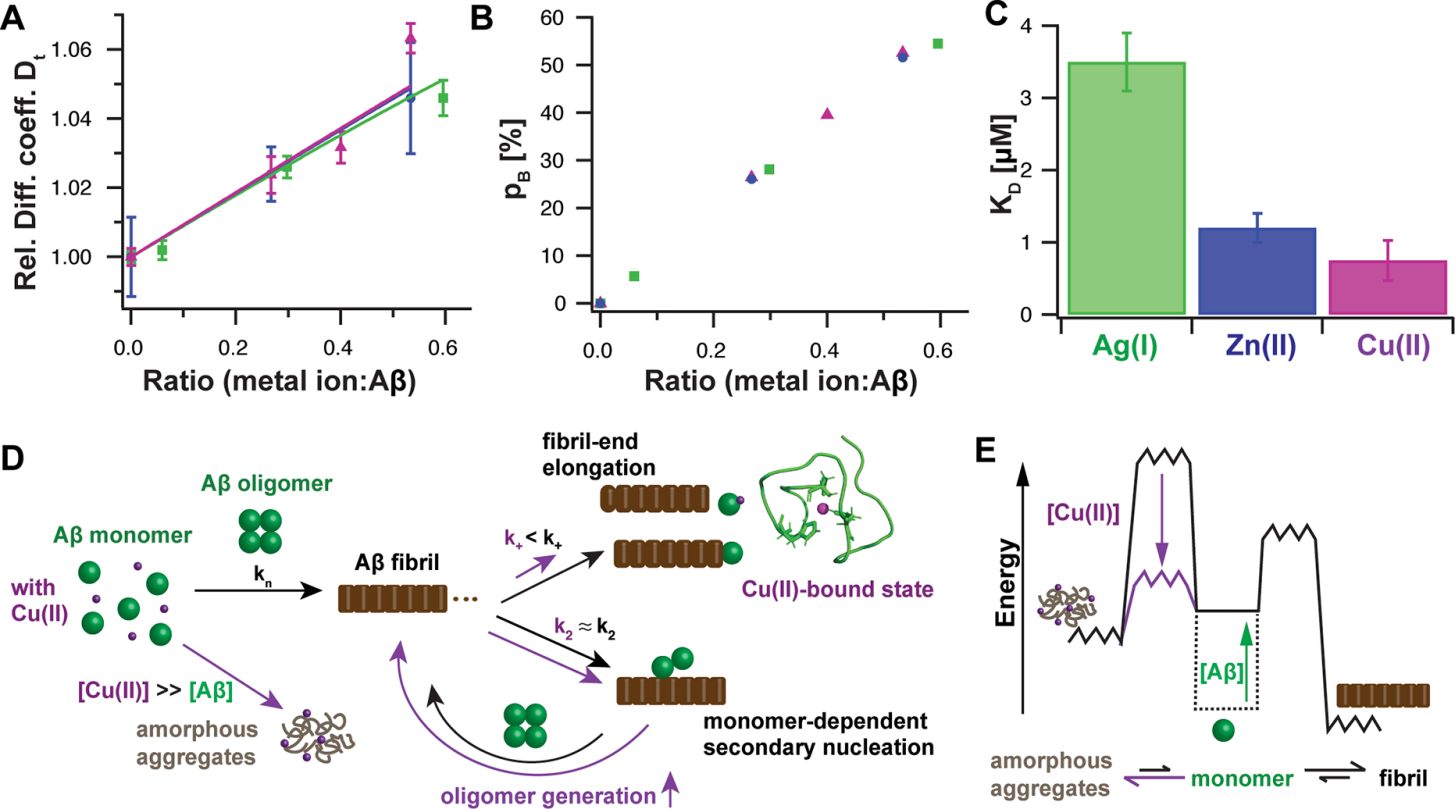
Model for Aβ inhibition mechanism of transition metal ions. (A) Global fit of the relative translational diffusion coefficients of Aβ40 in the presence of Ag(I) (green), Zn(II) (blue), or Cu(II) ions (violet) to a two-state model (free and bound state), resulting in a global fit parameter *D_B_*/*D*_free_ of 1.094 ± 0.005. (B) Population of the metal ion-bound state as determined from diffusion data in panel (A). (C) Apparent dissociation constant *K_D_* of Cu(II) binding to Aβ monomers from fit to relative elongation rates for Cu(II) (Figure 4H) and from previous results.^29^ (D) Schematic model for mechanism of action of inhibition of Aβ self-assembly by Cu(II). The metal ion-bound state is inert to aggregation, resulting in a predominate retardation of fibril-end elongation, which promotes the generation of new oligomers. (E) Free energy diagram visualizing the reaction scheme of amyloid fibrils formation, where increasing Aβ monomer concentration enhances the generation rate of amyloid formation. In contrast, high Cu(II) concentrations promote the formation of amorphous aggregates. Remarkably, this model is also qualitatively applicable to Zn(II) and Ag(I) ions, suggesting a common mechanism of action for these transition metal ions.

We have previously shown that both monovalent Ag(I) and divalent Zn(II) exhibit a similar mechanism of action both when binding to the N-terminus of Aβ and affecting Aβ fibrillization.^29^ Both metal ions form a dynamic complex with monomeric Aβ where the N-terminus encapsulates the metal ion. Comparing the metal ion-bound state to the case of Cu(II), we found that all these metal ions have a similar effect on the translational diffusion (Figure 6A). Indeed, the relative diffusion coefficient could be fitted globally to a two-state model consisting of a free and metal ion-bound state, revealing the same diffusion coefficient for the bound state, given by *D_B_*/*D*_free_ = 1.094 ± 0.005. In line with that, the populations of the bound state, *p_B_*, derived from the diffusion data, exhibit the same linear dependence on the metal ion to Aβ ratio (Figure 6B). This suggests that all investigated transition metal ions initiate a similar fold of the N-terminus wrapped around the metal ion and hence a qualitatively similar metal ion-bound structure.

Remarkably, comparable to Ag(I) and Zn(II),^25,29^ Cu(II) ions seem to primarily affect fibril-end elongation, hence suggesting a common mechanism of action of inhibition of Aβ fibrillization by these metal ions. In comparison to Ag(I) and Zn(II), Cu(II) has the highest binding affinity to Aβ monomers.^68,70^ However, as in the case for Cu(II), there are discrepancies present regarding the dissociation constant for Zn(II)-Aβ binding and it was previously demonstrated in competitive intrinsic tyrosine fluorescence experiments that Cu(II) quenches Y10 fluorescence of Zn(II)-bound Aβ,^30^ and also simultaneous Cu(II) and Zn(II) binding toward Aβ has been reported.^71,72^ Also here, we found that the apparent dissociation constant of Cu(II) to monomeric Aβ, which was derived from a fit to the relative elongation rates (Figure 4I), is smaller than for the other metal ions. In light of the broad range of dissociation constants reported in the literature, here the same model and similar experimental conditions have been used for a sound comparison. This leads to the order of dissociation constants of *K*_*D*_^app^(Cu(II)) < *K*_*D*_^app^(Zn(II)) < *K*_*D*_^app^(Ag(I)) to monomeric Aβ peptide (Figure 6C). Similar to Cu(II), high concentrations of Zn(II) were reported to result in amorphous aggregates,^21,22^ which thus is a shared feature particularly of divalent metal ions.

## Concluding Remarks

Taken together, we rationalized our findings in a model that provides a detailed understanding about the inhibition effect of Cu(II) binding to monomeric Aβ peptides on the Aβ self-assembly process, based on the NMR structure describing a molecular model of the Cu(II)-Aβ complex (Figure 6D). In this model, the Cu(II) ion is bound to the four binding ligands D1 (NH_2_ and CO), H6 (N_ε_), and H13 (N_δ_) or H14 (N_δ_), forming a well-structured fold in the first 23 N-terminal residues of Aβ. Our data showed that these four binding ligands are the preferential ones at pH 7.2, and alternative binding ligands could be excluded based on the structural constraints (Table 1). In addition, the combination of MD simulations with the structural constraints allowed us to identify the chirality mode of the metal complex formed upon D1 coordination.

Here, we propose that Cu(II) binding can obstruct the Aβ peptide to adapt a β-structure and thereby creating an aggregation-inert metal ion complex by mainly perturbing the fibril-end elongation nucleation event. In this perspective, the inhibition of Aβ aggregation can be understood by forming an apparently aggregation-inert Cu(II)-bound state, reducing the aggregation-prone Aβ monomer pool by the metal ion-bound population.

Remarkably, in the most recent molecular fibril structures reported in the literature, the N-terminus is completely^73,74^ or partly^75−78^ included in the fibril core of Aβ40 and Aβ42 fibrils. Hence, based on these reported structures also the N-terminus may be an essential part in the Aβ fibril structure. Due to the multistep reaction of the fibril-end elongation process,^79^ including a β-structure formation step in addition to the fibril attachment step, this nucleation event may be predominately affected by Cu(II). Indeed, primary and secondary nucleation do not necessarily involve the adaption of a β-structure and might, hence, be less sensitive to a N-terminal bound metal ion.

Notably, the specific inhibition of the elongation rate implies a greatly increased generation of Aβ oligomers as obtained from our kinetic analysis. This results may provide a deeper understanding how Aβ-induced cytotoxicity is modulated by Cu(II). In a recent study, the amount of generated oligomers could be quantified, enabling the application of more advance theoretical models including conversion rates from oligomers to fibrils.^80^ In future studies, this approach could be transferred to Cu(II)-modulated aggregation of Aβ including pathogenic mutations, which were reported to be affected differently by Cu(II).^28^

The strikingly similar binding and inhibition mechanism of Cu(II) compared to the previously reported Ag(I) and Zn(II) metal ions suggests then a general mechanism of action of these transition metal ions. All these metal ions form a metal-bound complex with monomeric Aβ, with different binding affinities, that apparently is inert to aggregation. This process might, thus, lead to a reduction of the aggregation-prone Aβ monomer pool, which inhibits Aβ self-assembly by predominately retarding fibril-end elongation – an evidently shared mechanism between these metal ions.

Further, the promotion of amorphous aggregate formation can be understood by a schematic energy diagram where high Cu(II) concentrations, similarly to high Zn(II) concentrations, lower the energy barrier toward amorphous aggregate, and make fibril formation less favorable (Figure 6E).

To conclude, our study provides high-resolution structural insights into metal ion-modulated Aβ aggregation kinetics *in vitro*. These findings may help to understand the Aβ aggregation behavior *in vivo* where metal ions, in particular Cu(II), may be one key environmental factor influencing the Aβ self-assembly process and Aβ-induced toxic effects.

## Materials and Methods

### Sample Preparation

For NMR, all experiments were performed on 75 μM ^15^N- or ^13^C-^15^N-labeled Aβ40 samples in 10 mM HEPES, pH 7.2–7.4, 10% D_2_O in the presence of different Cu(II) concentrations. Cu(II) ions were added as chloride salt. Aβ samples were always freshly prepared by initially dissolving the lyophilized peptide, purchased from AlexoTech (Umeå, Sweden), in 10 mM NaOH followed by sonication in a water-ice bath for ca. 1 min. Then, the sample was diluted to 10 mM final HEPES buffer concentration and sonicated for the same time again.^25,81^

For aggregation kinetics experiments, Aβ42 and Aβ40 peptides were expressed and purified as described in ref (82). In brief, Aβ was express with a designed solubility tag obtained from the N-terminal domain of a spider silk protein from flagelliform spidroins. After cell lysis, the fusion protein is dissolved in 8 M urea and purified using immobilized metal ion affinity chromatography, where the His tag of the fusion protein binds to the column. After elution and buffer exchange, the fusion protein is cleaved by TEV protease. Subsequently, monomeric Aβ is obtained after size exclusion chromatography. Monomeric Aβ peptides were used directly after the SEC step for aggregation kinetics experiments.

### NMR Experiments

^1^H-^15^N HSQC titration and ^1^H_N_-R_1_ and ^1^H_N_-R_2_ experiments were conducted with a ^15^N-labeled Aβ40 sample in 10 mM HEPES buffer at 281 K on a Bruker 950 MHz spectrometer, equipped with a TCI cryogenic probe. All NMR data was processed using Bruker Topspin or NMRPipe^83^ and analyzed with NMRFAM-Sparky^84^ and Matlab. ^1^H_N_-R_1_ longitudinal relaxation rates were obtained from 16 different delays from 0.002 to 2 s. The data was fitted to an exponential function:
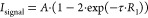
1

^1^H_N_-R_2_ transverse relaxation rates were recorded using 16 different delay times, τ, from 5 to 200 ms. The rate constants were obtained by fitting an exponential function, which is modulated by the *J*_HNHA_ coupling, described by
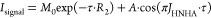
2

The coupling constant *J*_HNHA_ values were constrained to previously published values.^85^

The PRE values and the associated PRE distances were calculated as differences from ^1^H_N_-R_2_ rates without and with 100 μM Cu(II) as described in eq 3:
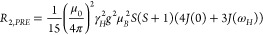
3where γ_*H*_ is the gyromagnetic ratio of ^1^H, *g* is the electron g-factor, ω_*H*_ is the Larmor frequency, and *S* is the spin number. The spectral density function, *J*(ω), is given by
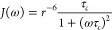
4in which the correlation time τ_*c*_ is defined as τ_*c*_ = (τ_*r*_^–1^ + τ_*s*_^–1^), where τ_*r*_ is the rotational correlation time of the macromolecule and τ_*s*_ is the effective electron relaxation time. Here, we used τ_*s*_ = 5.0 and τ_*r*_ = 2.56 ns as the mean correlation time of Aβ40 at 281 K,^86^ giving a correlation time of τ_*c*_ = 1.69 ns. The obtained PRE distances are summarized in Table S1.

Also, ^1^H-^15^N paraHSQC, using specially designed pulse sequences for recovering fast relaxing resonances,^52^ was performed on the ^15^N-labeled Aβ40 sample on the Bruker 950 MHz spectrometer..

Direct ^13^C-detected paramagnetic experiments were conducted with a ^13^C-^15^N-labeled Aβ40 sample on a Bruker 700 MHz spectrometer equipped with a TXO cryogenic probe, which is sensitivity-improved for direct ^13^C detection. Paramagnetic CON, CaCO and CbCaCO experiments, which were tailored to detect fast relaxing resonances.^52^ The spectra were assigned using literature values.^85^

NMR diffusion experiments were performed on 75 μM Aβ40 with and without different concentrations of Cu(II) at 281 K on a 700 MHz Bruker spectrometer with cryogenic probe. A list of 16 different gradient strengths using a gradient pulse length of 5 ms and diffusion time of 150 ms was applied. The diffusion coefficients were obtained by integrating the signals of the methyl groups. The hydrodynamic radius was calculated from the diffusion coefficient *D_t_* using the Stokes–Einstein equation:
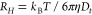
where *k*_B_ is Boltzmann’s constant and η the dynamic viscosity. Errors are given as standard deviations of 10 different measurements.

### Structure Calculation

The structural calculations were performed for the first 23 N-terminal residues using CYANA^54^ where the lower and upper distance limits were applied as listed in Table S2. The calculations were performed using 10,000 starting structures and the outcome of the best five from 100 selected conformers. For the PRE constraints, a distance interval of ± 0.5 Å was applied. The metal ion was linked to the nitrogen of the NH_2_-terminus, the amide oxygen of D1, and the N_ε_ of H6 and N_δ_ of H13 or H14. Structural refinement was conducted using the AMBER 20 package.^87^

### MD Simulations

The two structural models with H13 or H14 as the fourth ligand obtained from CYANA calculations were used as input for MD simulations. In the MD simulations, we also took into account that the formation of the metallacycle by Cu(II) coordination to the NH_2_ terminus and the backbone CO group from D1 generates two possible chiral conformers. Thus, we performed four different MD simulations starting from four different structures. A detail description is given in the Supporting Information.

### Aggregation Kinetics

ThT aggregation kinetics experiments were conducted on a FLUOStar Galaxy (BMG Labtech) fluorometer using 384 well plates with 20 μL solution per well. Aggregation traces on 3 μM Aβ42, in 20 mM sodium-phosphate buffer, pH 8, during quiescent conditions, with 10 μM ThT and different concentrations of Cu(II) were recorded in replicates of five. Experiments were repeated ≥5 time, using ≥3 different peptide batches from different purifications, giving qualitatively similar results. For Aβ40, 20 μM Aβ40 in 20 mM sodium-phosphate buffer, pH 7.2, was used and 6 replicates were recorded. A sigmoidal function was fitted to the aggregation traces to extract the aggregation half time. Further, after averaging and normalization of the aggregation traces, the kinetic data was fitted with a kinetic nucleation model^15,61^ (equations are given in the Supporting Information). In this model, there are free fitting parameter, represented by the combined rate constants  and . First, we performed an individual fit to each aggregation trace to obtain the dependence of these parameters on the Cu(II) concentration. Second, we fitted the aggregation data globally, where one of the fitting parameters was constrained to the same value across all Cu(II) concentrations. With this constrain, only one nucleation rate constant is the sole free fitting parameter, enabling us to test whether modulation in only one nucleation rate constant can adequately describe the kinetic data. The fitting procedure was performed with IgorPro software and equations previously published.^15,18^

Highly seeded aggregation kinetic experiments were performed with 3.2 μM Aβ42 in the presence of ca. 1.5 μM seeds (pre-formed sonicated fibrils). The elongation rate was obtained by linear fits to the first 9 min, neglecting the very first points due to an equilibration phase.

### Apparent Dissociation Constant from the Relative Fibril-End Elongation Rate

Assuming that the Cu(II)–bound Aβ complex is aggregation-inert, which implements that the metal ion-bound Aβ peptides are removed from the aggregation-prone peptide pool, the effect of Cu(II)-binding to Aβ monomers on the elongation rate can be described in terms of an apparent dissociation constant *K_D_* by^64^
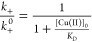
5where *k*_+_/*k*_+_^0^ refers to the relative elongation rate. Fits were performed on the combined data sets from the global fit analysis (Figure 4F) and highly seeded aggregation kinetics (Figure 4G). Error analysis was performed using fits on the error-weighted data (lower gray curve in Figure 4H) and outliers-adjusted data (upper gray curve in Figure 4H, where the three values with the larges errors were removed).

### Estimation of Oligomer Generation from Kinetic Analysis

The number of new nucleation units can be calculated from the integral over the reaction profile.^17,18,20^ A detailed description is given in the Supporting Information.

### TEM Analysis

5 μL of Aβ42 fibrils with or without Cu(II), obtained directly after the aggregation kinetics experiments, was spotted on formvar/carbon coated 400 mesh copper TEM grids. After 10 min, the excess sample was removed with blotting paper. Then, the grid was washed twice with MQ water followed by staining with 1% aqueous uranyl acetate solution for 5 min. The grid was air-dried and then TEM imaging (FEI Tecnai 12 Spirit BioTWIN, operated at 100 kV) was performed. Images were recorded using a 2 k × 2 k Veleta CCD camera (Olympus Soft Imaging Solutions, GmbH, Münster, Germany). Images were recorded at magnification of 43,000×.

## Supporting Information Available

Additional method description, NMR spectra, NMR relaxation data, MD simulation results, Aβ40 kinetics results, and a complete table of structural constraints and parameters of structure determination (PDF)

## Notes

The atomic coordinates, chemical shifts and structural restraints have been deposited in the Protein Data Bank with the PDB ID codes 8B9Q and 8B9R for H13 and H14 as fourth ligand, respectively, and the Biological Magnetic Resonance Bank with BMRB accession numbers 34762 and 34763 for H13 and H14 as fourth ligand, respectively.
