# Supplementary material for: Molecular Structure of Cu(II)-Bound Amyloid-β Monomer Implicated in Inhibition of Peptide Self-Assembly in Alzheimer’s Disease
Source: JACS Au. 2022 Nov 11;2(11):2571–84. doi: 10.1021/jacsau.2c00438 (PMC9709942; doi:10.1021/jacsau.2c00438)
Supplement: Supplementary file 1 — au2c00438_si_001.pdf [file au2c00438_si_001.pdf]

Supporting Information

Molecular structure of Cu(II)-bound amyloid-β monomer implicated in inhibition of peptide self-assembly in Alzheimer's

Axel Abelein[1,*], Simone Ciofi-Baffoni[2], Cecilia Mörman[1,3], Rakesh Kumar[1], Andrea Giachetti[2], Mario Piccioli[2], Henrik Biverstål[1,4,*]

[1] Department of Biosciences and Nutrition, Karolinska Institutet, 141 83 Huddinge, Sweden

[2] Magnetic Resonance Center and Department of Chemistry, University of Florence, Via Luigi Sacconi 6, 50019 Sesto Fiorentino, Florence, Italy

[3] Department of Biochemistry and Biophysics, The Arrhenius Laboratories, Stockholm University, 106 91, Stockholm, Sweden

[4] Department of Physical Organic Chemistry, Latvian Institute of Organic Synthesis, Riga LV-1006, Latvia

Amyloid-β peptide, Alzheimer's disease, copper ion, paramagnetic NMR, aggregation kinetics

SUPPORTING INFORMATION METHODS

MD simulations

The systems were inserted on truncated octahedral box of TIP3P water molecules extending to at least 10 Å from the protein atoms[1]. Amber force field Ff19SB was used for the protein[2] and the force field parameters of Cu(II) bound to H6, H13 (or H14), and D1 was adapted from Ref. [3]. In particular, the chirality *a* mode was obtained by changing the angles of N and O atoms on D1. The Cu atom was set to coordinate the backbone atoms N and O of D1, side-chain atom N_ε of H6 and side-chain atom N_δ for H13 or H14. The MD simulations were carried out using AMBER 20 package[4,5]. The Leapfrog algorithm[6] was used for equations of motion that were integrated using a time step of 2 fs. The SHAKE algorithm[7] was used to constrain the length of all bonds related to hydrogen atoms. Particle mesh Ewald method[8] was employed to treat the long-range electrostatic interactions. The systems were first subjected to initial minimization followed by warming up to 300K in 100 ps using NVT ensemble. The final trajectory was carried out using NPT ensemble for a total of 1.3 μs, and the last 1 μs were used for the data analysis.

Kinetic Analysis

For the kinetic analysis a nucleation model was applied[9,10] that includes the nucleation events primary and secondary nucleation, described the nucleation rate constants k_n and k_2, in addition to fibril-end elongation with the rate constant k_+. For the fitting procedure two fitting parameters $\lambda = \sqrt{2 \cdot k_n k_+ \cdot m(0)^{n_C}}$ and $\kappa = \sqrt{2 \cdot k_+ k_2 \cdot m(0)^{n_2+1}}$ are defined, where $m(0)$ is the initial Aβ monomer concentration and n_C and n_2 the reaction orders for primary and secondary nucleation, respectively. From previous analysis these reaction orders are set to $n_C = n_2 = 2$.

The time dependence of the fibril mass $M(t)$ is then given by:

$$\frac{M(t)}{M(\infty)} = 1 - \left(\frac{B_+ + C_+}{B_+ + C_+ \cdot exp(\kappa t)} \cdot \frac{B_- + C_+ \cdot exp(\kappa t)}{B_- + C_+}\right)^{\frac{k_\infty^2}{\kappa \tilde{k}_\infty}} \cdot exp(-k_\infty t)$$

where the additional coefficients are functions of λ and κ:

$$C_\pm = \pm \lambda^2 /2/\kappa^2$$

$$k_\infty = \sqrt{2\kappa^2/(n_2(n_2+1)) + 2\lambda^2/n_C}$$

$$\tilde{k}_\infty = \sqrt{k_\infty^2 - 4C_+C_-\kappa^2}$$

$$B_\pm = (k_\infty \pm \tilde{k}_\infty)/2/\kappa$$

Generation of new nucleation units

From this model the time evolution of the rate of generation of new fibrils via nucleation units (which refer to low molecular weight on-pathway oligomers) can be described the nucleation rate $r_n(t)$ given by[11]:

$$r_n(t) = k_n m(t)^{n_C} + k_2 M(t) m(t)^{n_2}$$

The number of new nucleation units (oligomers) formed is then given by the integral over the complete reaction, described by the nucleation rate $r_n(t)$.

SUPPORTING INFORMATION FIGURES

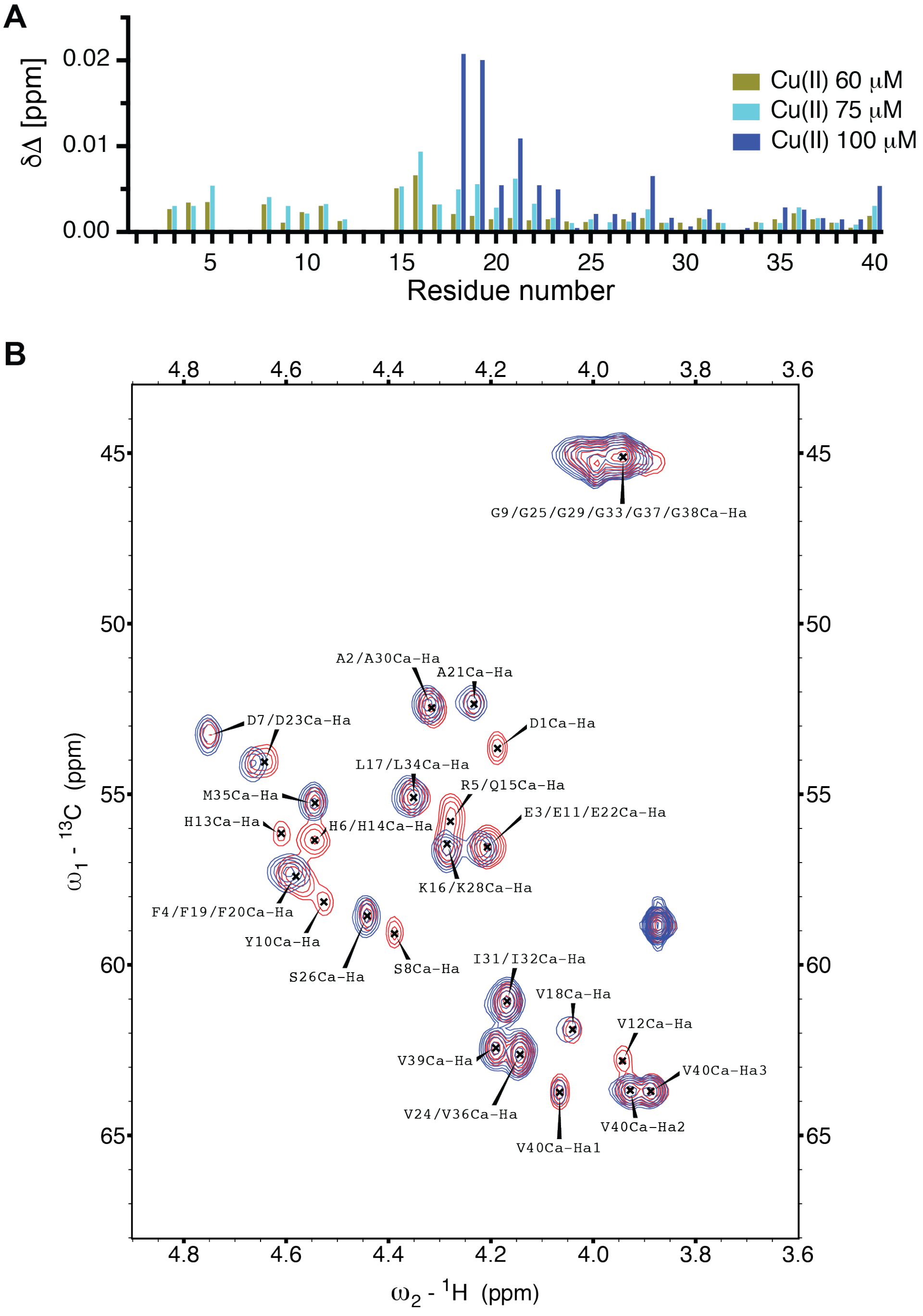

Supporting Information Figure S1. Chemical shift changes of ^{1}H-^{15}N HSQC and ^{1}H-^{13}C HSQC spectrum. (A) Combined chemical shift changes Δδ of ^{1}H-^{15}N HSQC from spectra in Figure 1 at different Cu(II) concentrations. Significant chemical changes are observed at 100 μM Cu(II) for residue 18 to 21, which are the still visible residues closest to the N-terminal binding site. **(B)** C^{α} region of ^{1}H-^{13}C HSQC spectrum without (red) and with 100 μM Cu(II), exhibiting a drastic signal loss of N-terminal residues including D1.

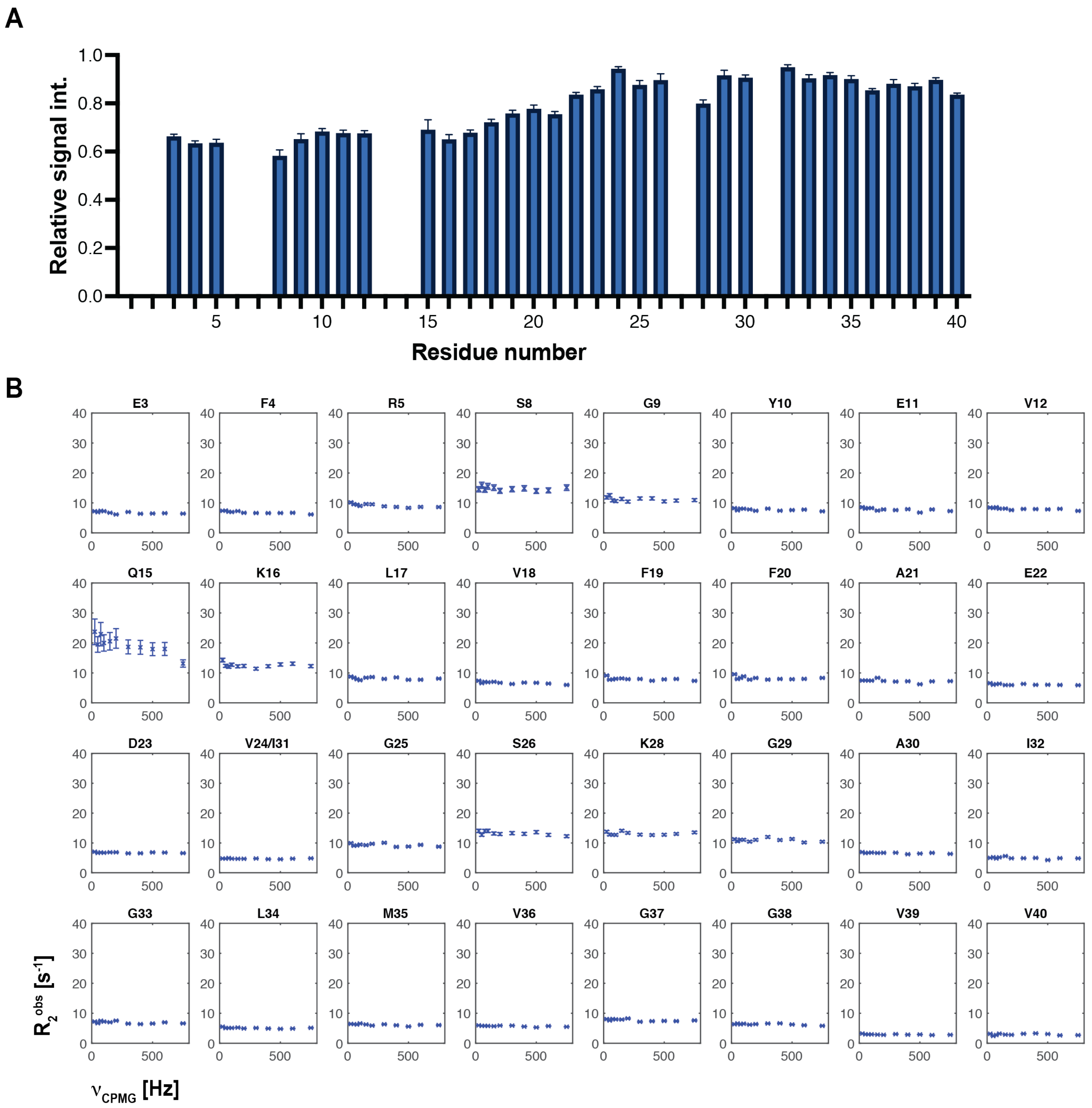

Supporting Information Figure S2. ^{15}N-CPMG relaxation dispersion experiments. Relaxation dispersion experiment were performed on 75 µM Aβ40 in 10 mM HEPES, pH 7.4, at 281 K in the presence of 20 µM Cu(II) ions at 700 MHz. **(A)** ^{1}H-^{15}N HSQC cross-peak intensities are displayed, showing a signal attenuation of the N-terminal residues. **(B)** ^{15}N-CPMG relaxation dispersion experiments recorded at 11 different CPMG frequencies exhibit flat relaxation dispersion profiles.

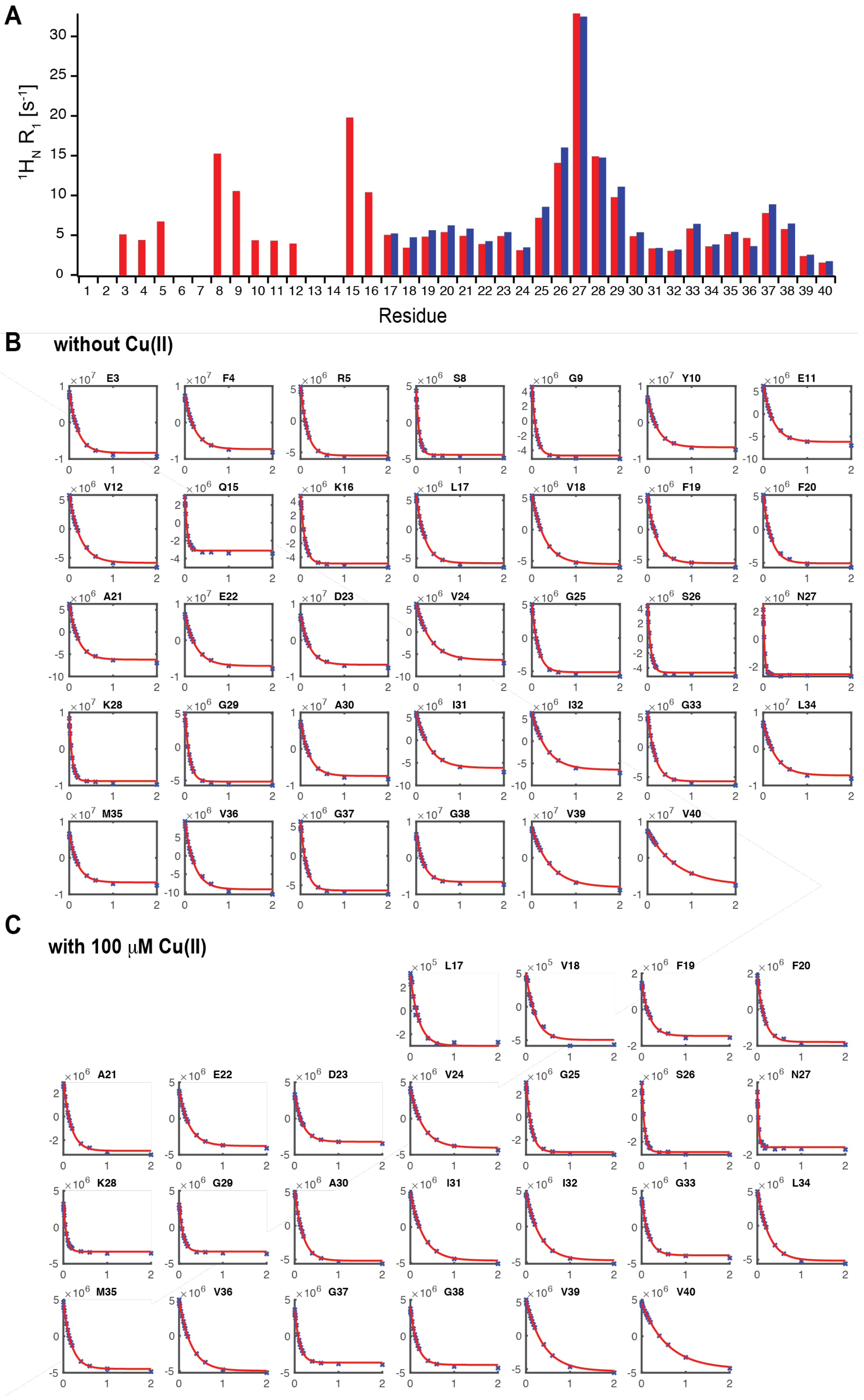

Supporting Information Figure S3. ^1H_N-R_1 longitudinal relaxation rates. (A) ^1H_N-R_1 were obtained from fits to signal intensities at 16 different delay times without (red) and with 100 µM Cu(II) (blue) using Eq. (1). **(B,C)** The fits to the relaxation profiles are displayed in (B) without and in (C) with 100 µM Cu(II),

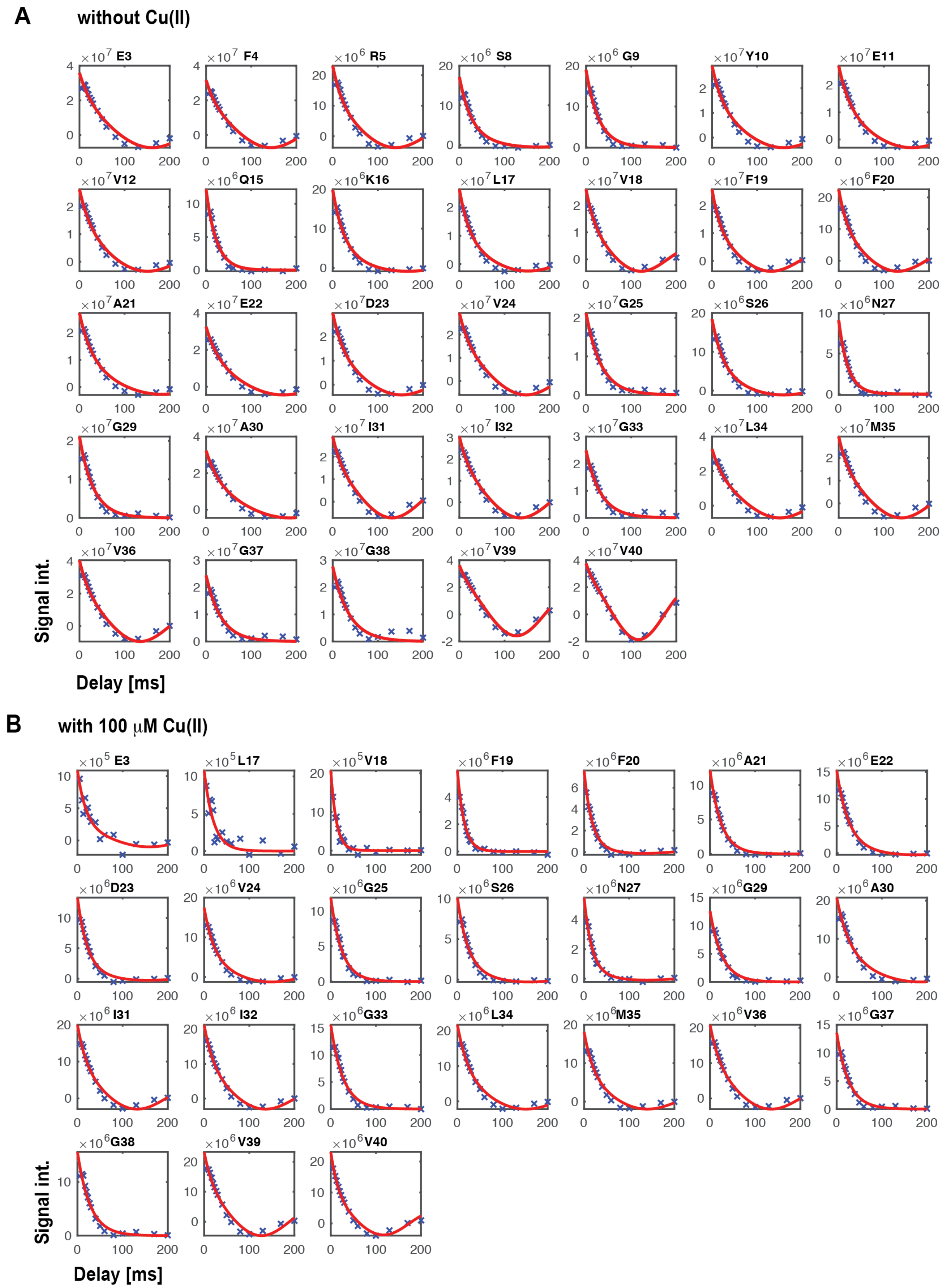

Supporting Information Figure S4. ^1H_N-R_2 transverse relaxation rates. ^1H_N-R_2 were obtained from fits to signal intensities at 16 different delay times without (A) and with 100 µM Cu(II) using equation Eq. (2) accounting for the coupling constant J_{HNHA}. The values for the coupling constant were obtained from Ref. [12].

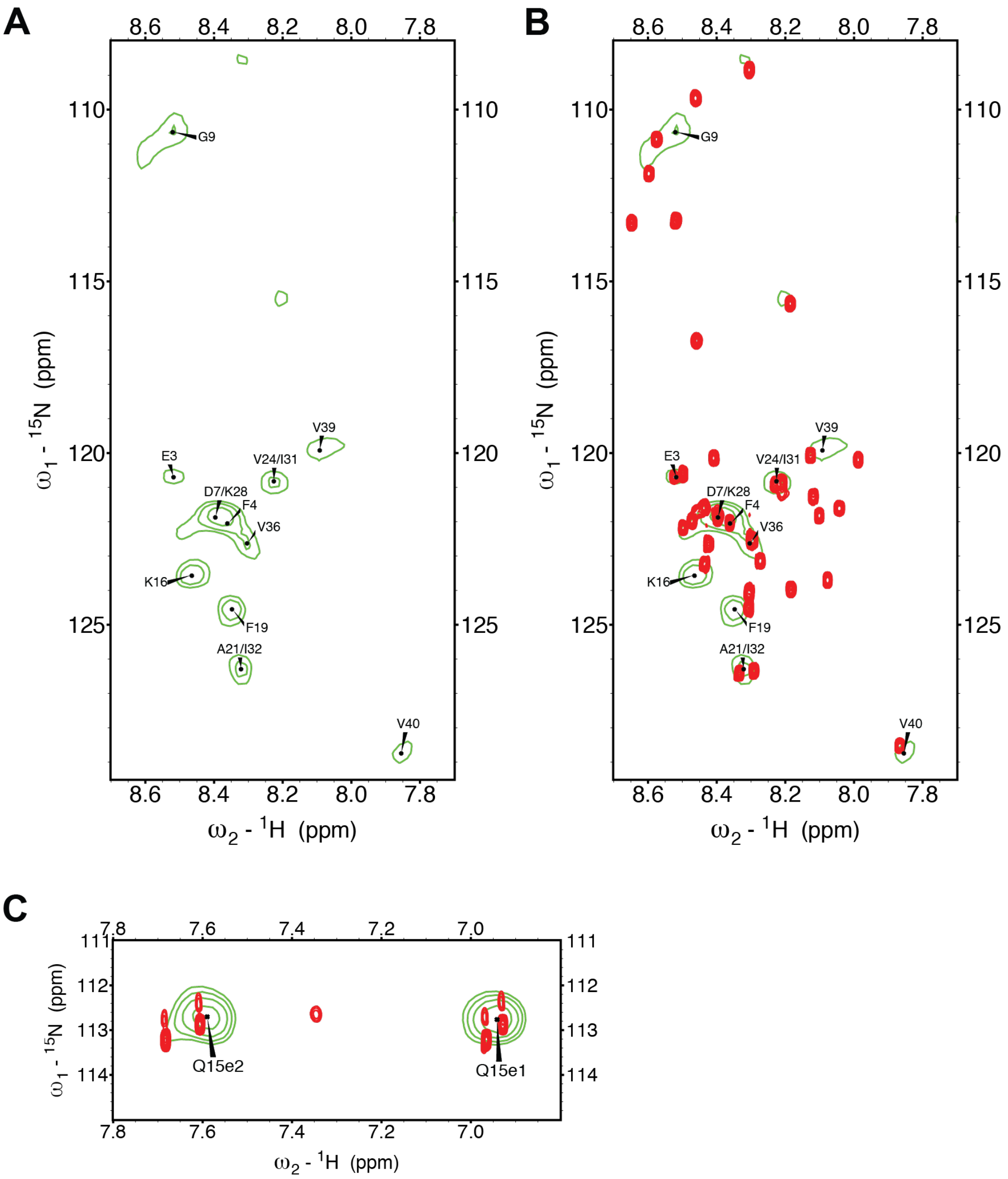

Supporting Information Figure S5. Paramagnetic HSQC experiments. (A) Optimized paramagnetic IR HSQC experiments on 75 μM Aβ40 in 10 mM HEPES, pH 7.2 in the presence of 100 μM Cu(II). (B) Overlay of paraHSQC experiment in the presence of 100 μM Cu(II) (green) and diamagnetic ^{1}H-^{15}N HSQC without Cu(II) (red). (C) Overlay of side chain region.

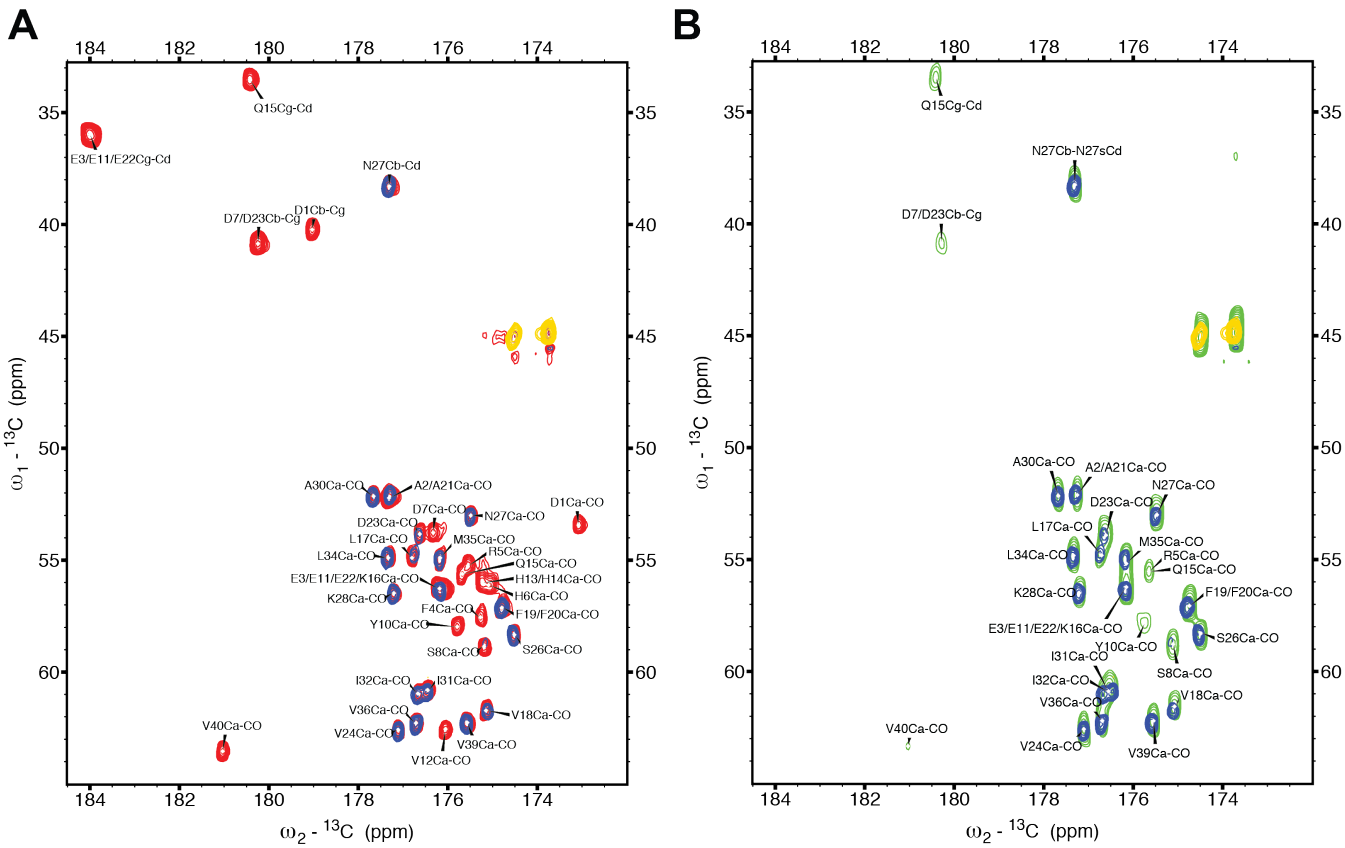

Supporting Information Figure S6. Paramagnetic CaCO experiments. (A) Overlay of diamagnetic CaCO experiments on 75 µM Aβ40 in 10 mM HEPES, pH 7.2 without (red) and with 100 µM Cu(II) (blue). (B) Overlay of diamagnetic (blue) and paramagnetic (green) experiment in the presence of 100 µM Cu(II). Negative cross-peaks are colored in yellow.

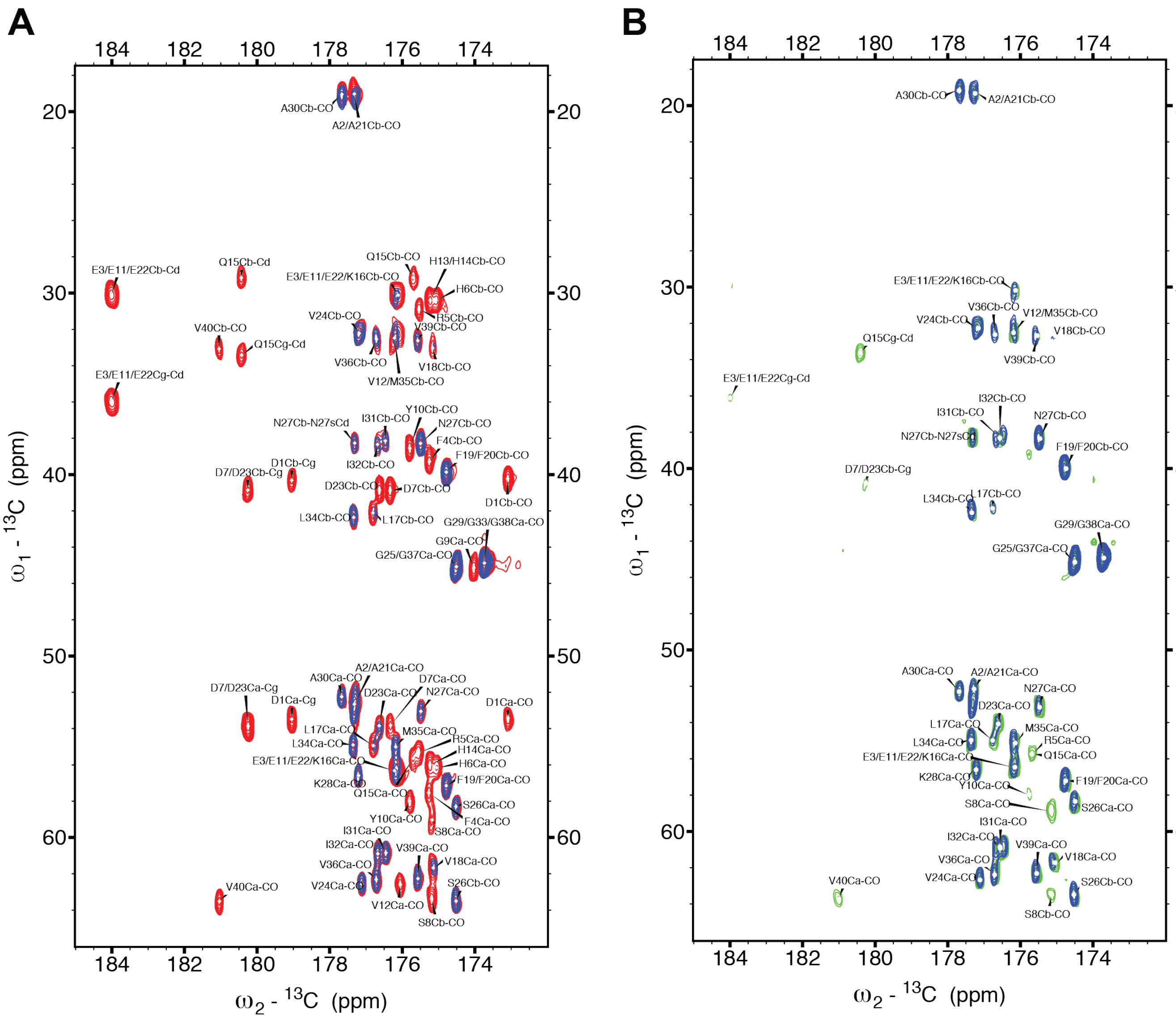

Supporting Information Figure S7. Paramagnetic CbCaCO experiments. (A) Overlay of diamagnetic CbCaCO experiments on 75 μM Aβ40 in 10 mM HEPES, pH 7.2 without (red) and with 100 μM Cu(II) (blue). (B) Overlay of diamagnetic (blue) and paramagnetic (green) CbCaCO experiment in the presence of 100 μM Cu(II).

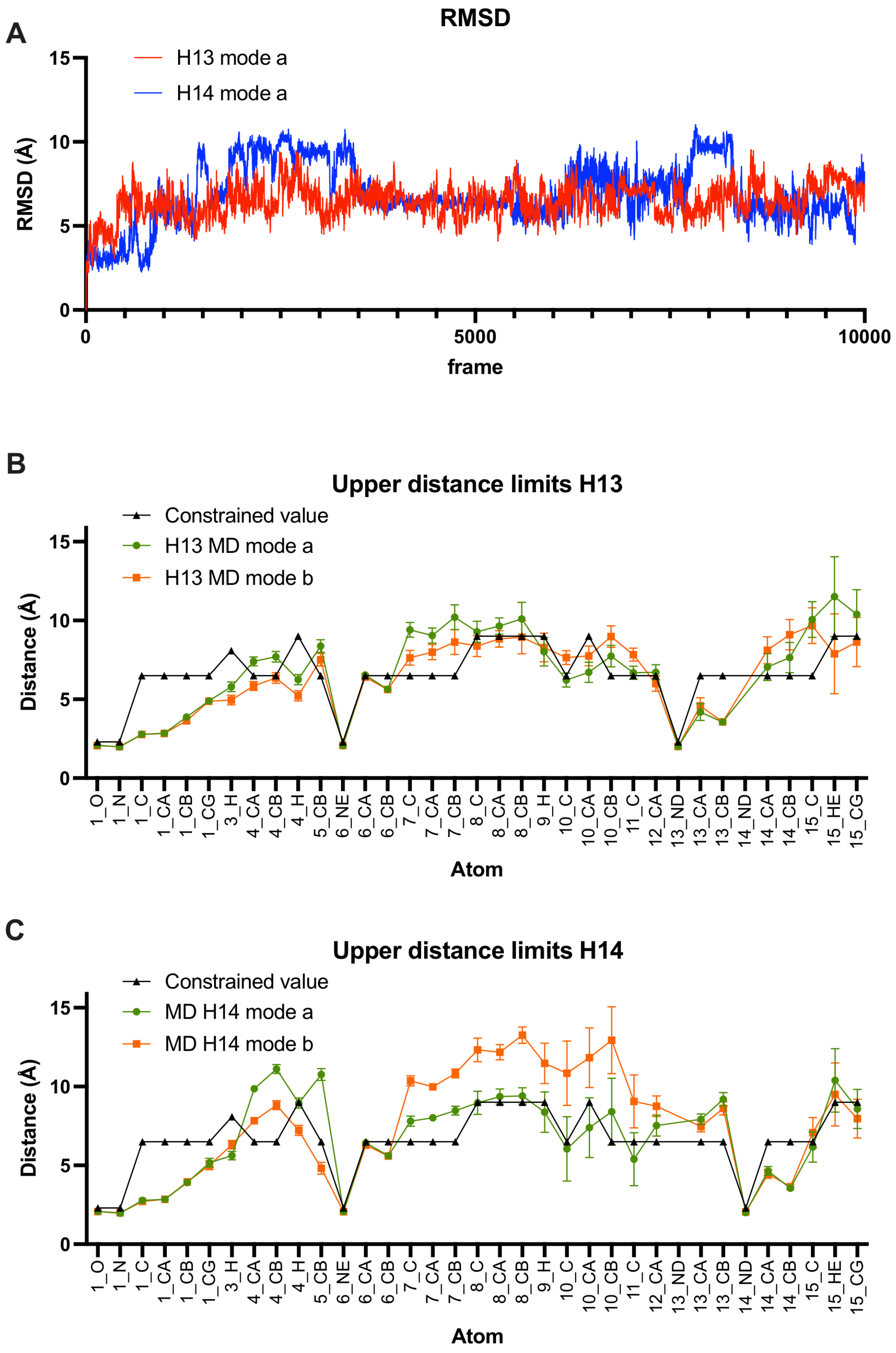

Supporting Information Figure S8. MD simulations on Cu(II)-bound Aβ40 complex. (A) Time dependence of RMSD value for chirality mode *a* with H13 (red) and H14 (blue) as fourth binding ligand, demonstrating the stability of the structures. **(B,C)** Upper distance constraints of experiments (black) and MD simulations for chirality mode *a* (green) and *b* (orange) for H13 (B) and H14 (C) as fourth binding ligand, respectively. These results indicate a better agreement of chirality mode *a* with the experimental constraints.

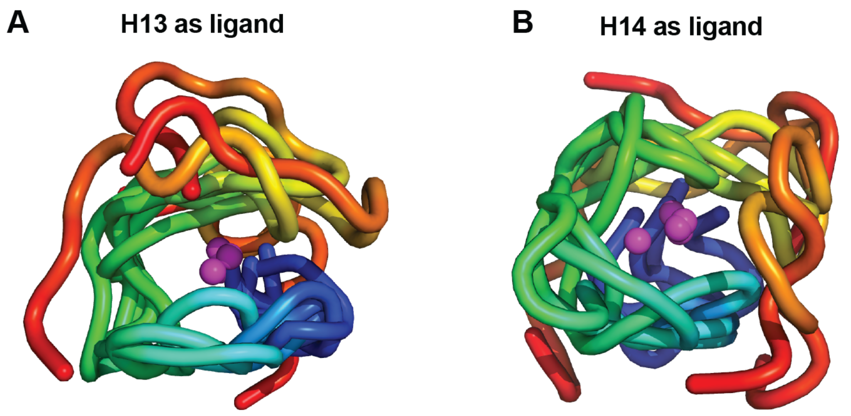

Supporting Information Figure S9. Structural ensemble of five best conformers of Cu(II)-Aβ complex. The five best conformers for chirality mode *a* are visualized after AMBER refinement for H13 (A) and H14 (B) as forth ligand, respectively.

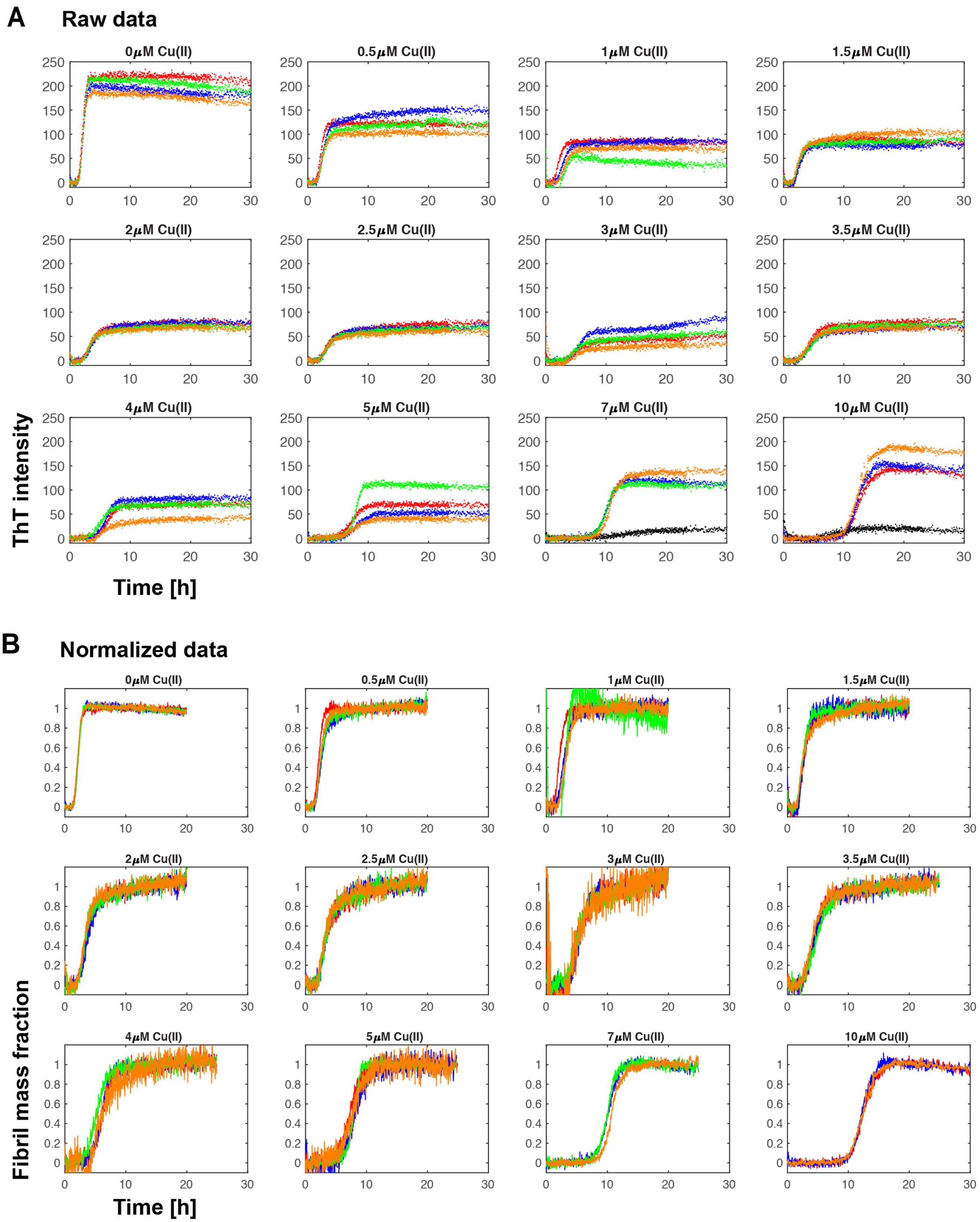

Supporting Information Figure S10. Raw data and normalized data of Aβ42 aggregation kinetics. (A) Raw data of ThT aggregation kinetics using four replicates. Apparent outliers are plotted in black color, which are neglected for normalization and averaged kinetic traces visualized in Figure 4. **(B)** Normalized individual aggregation traces.

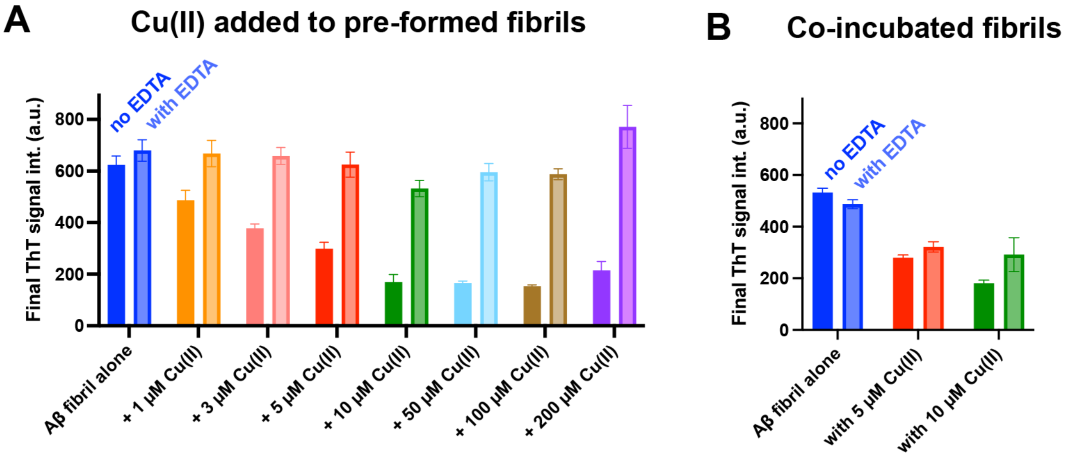

Supporting Information Figure S11. Final ThT signal intensity of pre-formed and co-incubated Aβ42 fibrils with Cu(II). (A) ThT final intensity of pre-formed Aβ42 fibrils with addition of different concentrations of Cu(II) and subsequent addition of 5 mM EDTA. **(B)** ThT final intensity of Aβ42 fibrils produced by co-incubation of Aβ42 monomers with different concentrations of Cu(II), and subsequent addition of 10 mM EDTA.

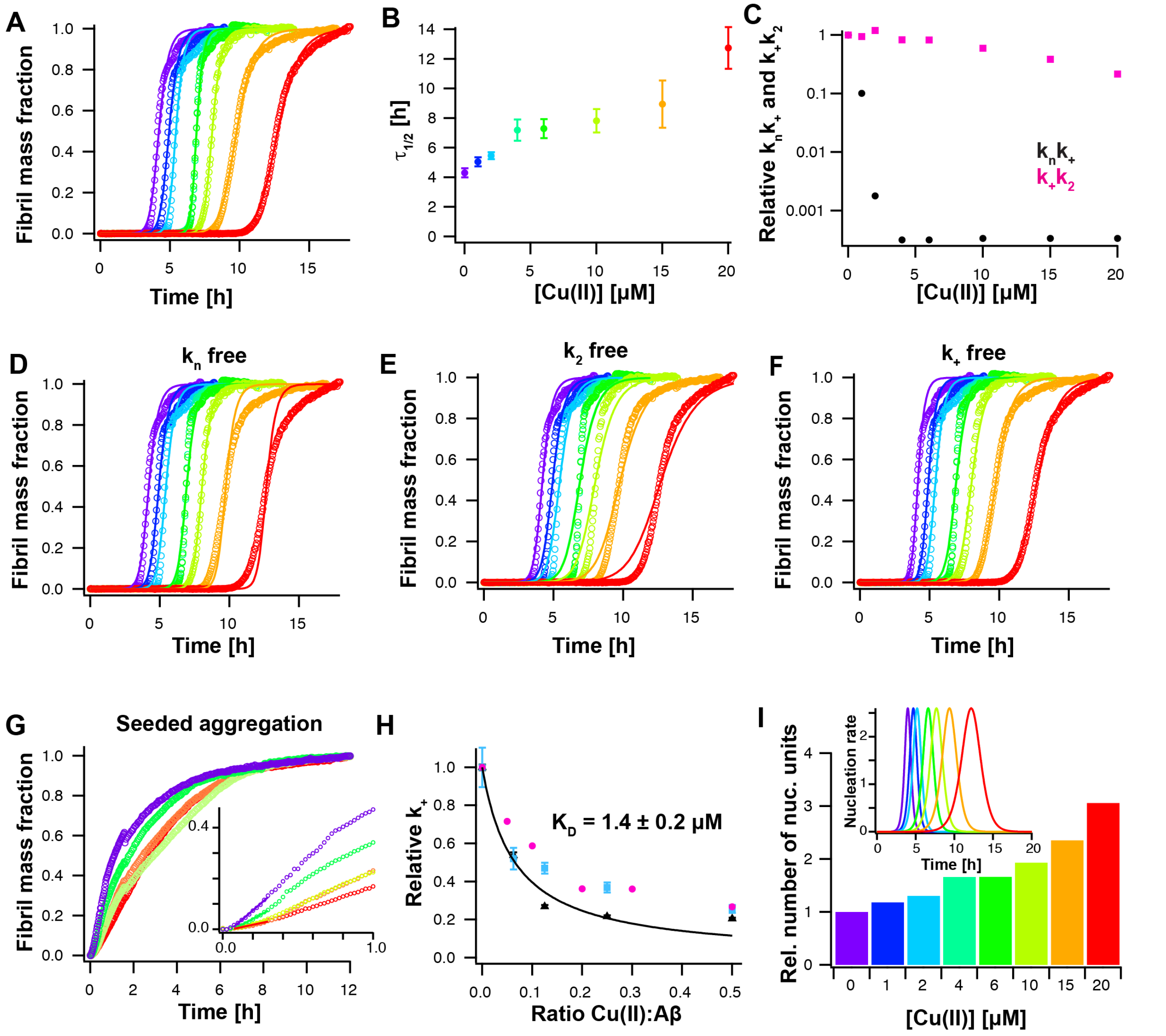

Supporting Information Figure S12. Analysis of aggregation kinetics of Aβ40 reveals specifically retarding fibril-end elongation. (A) Aggregation kinetics of 20 µM Aβ40 in 20 mM sodium-phosphate buffer, pH 7.2, in the presence of different Cu(II) concentrations from 0 (violet) to 20 µM Cu(II) (red). The aggregation traces were individually fitted with a nucleation model, including primary and secondary nucleation as well as fibril-end elongation. **(B)** Aggregation half times, $\tau_{1/2}$, obtained from sigmoidal fits of the aggregation traces in (A). **(C)** Relative combined rate constant $k_n k_+$ (black) and $k_+ k_2$ (pink), related to primary and secondary nucleation processes, respectively, as obtained from individual fits constrained to decreasing $k_n k_+$ values with increasing Cu(II). **(D-F)** Global fit analysis of aggregation traces where the fit parameters were constrained such that only one nucleation rate constant is the sole fitting parameter, *i.e.* k_n in (D), k_2 in (E) and k_+ in (F). The χ^2 values are 3.63, 6.70 and 2.49 for k_n, k_2 and k_+, respectively, revealing the best fit for k_+. **(G)** Highly seeded aggregation kinetics of 20 µM Aβ40 in 20 mM sodium-phosphate buffer, pH 7.2, in the presence of 1 µM pre-formed seeds, where the initial slope reflects the relative elongation rate. The inserted graph is a zoom with the linear fits of the first 1 h. **(H)** Fit of the relative elongation rates from ThT data plotted against the Cu(II):Aβ40 ratio reveals an apparent dissociation constant of $K_D^{app} = 1.4 \pm 0.2$ µM. Relative elongation rates obtained from seeded aggregation assays using ThT (black) and pFTAA (light blue) binding dyes as well as from global fit analysis (pink) giving qualitatively similar results. **(I)** Relative number of nucleation units at different Cu(II) concentrations obtained from the integral of the nucleation rate (inserted graph). The nucleation rate is calculated from the parameters of the kinetic analysis and given in units of M s^{-1} x10^{-13}.

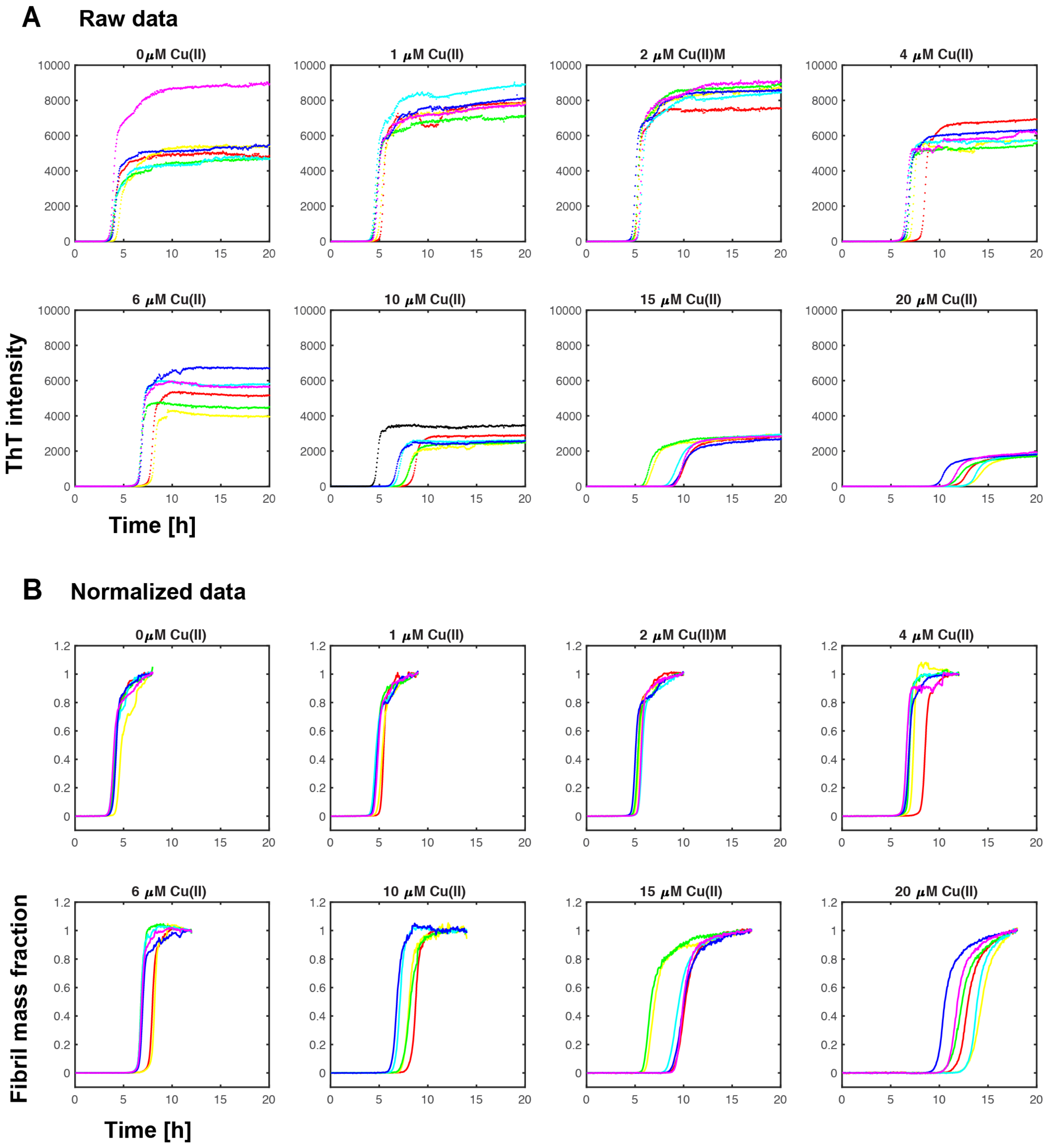

Supporting Information Figure S13. Raw data and normalized data of Aβ40 aggregation kinetics. (A) Raw data of ThT aggregation kinetics using six replicates. Apparent outliers are plotted in black color, which are neglected for normalization and averaged kinetic traces visualized in SI Figure S12. **(B)** Normalized individual aggregation traces.

SUPPORTING INFORMATION TABLES

Supporting Information Table S1. PRE distances from transverse $^{1}H_{N}$-R_2 relaxation rate measurements. The transverse $^{1}H_{N}$-R_2 relaxation rate were obtained for 75 µM Aβ40 with and without 100 µM Cu(II) where the PRE corresponds to the difference. Using Eq. (3) and (4) in the main article the PRE radius can be calculated.

Residue	PRE radius [Å]
3	7.59
17	8.49
18	6.75
19	7.45
20	7.93
21	8.57
22	9.10
23	8.79

Supporting Information Table S2. Structural constraints used in CYANA calculation based on diverse paramagnetic experiments.

Experiment	Residue no	Residue	Atom	LOL [Å]	UPL [Å]	Note
Linked ligands						
Ligand	1	ASP	N	1.80	2.30	
Ligand	1	ASP	O	1.80	2.30	
Ligand	6	HIS	NE2	1.80	2.30	
Ligand	13	HIS	ND1	1.80	2.30	for component Ia
Ligand	14	HIS	ND1	1.80	2.30	for component Ib
PRE experiments						
PRE	3	GLU	H	7.09	8.09	PRE ± 0.5 Å
PRE	17	GLN	H	7.99	8.99	PRE ± 0.5 Å
PRE	18	VAL	H	6.25	7.25	PRE ± 0.5 Å
PRE	19	PHE	H	6.95	7.95	PRE ± 0.5 Å
PRE	20	PHE	H	7.43	8.43	PRE ± 0.5 Å
PRE	21	ALA	H	8.07	9.07	PRE ± 0.5 Å
PRE	22	GLU	H	8.60	9.60	PRE ± 0.5 Å
PRE	23	ASP	H	8.29	9.29	PRE ± 0.5 Å
paraHSQC experiments						
paraHSQC	3	GLU	H	6.50	9.00	
paraHSQC	4	PHE	H	6.50	9.00	
paraHSQC	9	GLY	H	6.50	9.00	
paraHSQC	16	LYS	H	6.50	9.00	
paraHSQC	19	PHE	H	6.50	9.00	
paraHSQC	15	GLN	HE2	6.50	9.00	
paraCON experiments						
paraCON	8	SER	C	4.50	9.00	
paraCON	16	LYS	C	4.50	9.00	
paraCON	17	GLN	C	4.50		also partly present in dia
paraCON	1	ASP	C		6.50	not recovered in para
paraCON	7	ASP	C		6.50	not recovered in para
paraCON	10	TYR	C		6.50	not recovered in para
paraCON	11	GLU	C		6.50	not recovered in para
paraCON	15	GLN	C		6.50	not recovered in para
paraCaCO experiments						
paraCaCO	8	SER	CA	4.50	9.00	
paraCaCO	10	TYR	CA	4.50	9.00	
paraCaCO	15	GLN	CG	4.50	9.00	
paraCaCO	1	ASP	CA		6.50	not recovered in para
paraCaCO	1	ASP	CB		6.50	not recovered in para

paraCaCO	4	PHE	CA		6.50	not recovered in para
paraCaCO	7	ASP	CA		6.50	not recovered in para
paraCaCO	12	VAL	CA		6.50	not recovered in para
paraCbCaCO experiments						
paraCbCaCO	8	SER	CA	4.50	9.00	
paraCbCaCO	8	SER	CB	4.50	9.00	
paraCbCaCO	10	TYR	CA	4.50	9.00	
paraCbCaCO	15	GLN	CG	4.50	9.00	
paraCbCaCO	17	LEU	CB	4.50	9.00	
paraCbCaCO	1	ASP	CA		6.50	not recovered in para
paraCbCaCO	1	ASP	CB		6.50	not recovered in para
paraCbCaCO	1	ASP	CG		6.50	not recovered in para
paraCbCaCO	4	PHE	CA		6.50	not recovered in para
paraCbCaCO	4	PHE	CB		6.50	not recovered in para
paraCbCaCO	5	ARG	CB		6.50	not recovered in para
paraCbCaCO	6	HIS	CA		6.50	not recovered in para
paraCbCaCO	6	HIS	CB		6.50	not recovered in para
paraCbCaCO	7	ASP	CA		6.50	not recovered in para
paraCbCaCO	7	ASP	CB		6.50	not recovered in para
paraCbCaCO	10	TYR	CB		6.50	not recovered in para
paraCbCaCO	13	HIS	CA		6.50	not recovered in para
paraCbCaCO	13	HIS	CB		6.50	not recovered in para
paraCbCaCO	14	HIS	CA		6.50	not recovered in para
paraCbCaCO	14	HIS	CB		6.50	not recovered in para

Supporting Information Table S3. Parameters from structure determination for Cu(II)-Aβ with H13 and H14 as fourth ligand, respectively.

**Parameters**	**Cu(II)-Aβ with H13 as ligand**	**Cu(II)-Aβ with H14 as ligand**
Distance restraints (total)	82	82
Upper limits distance restraints	53	53
Lower limint Distance restraints	29	29
Medium Range distance restraints 1 < (I -J) < 5	4 (upl) 15 (lol)	4 (upl) 15 (lol)
Intraresidual (|i - j| = 0)	8 (upl) 2 (lol)	8 (upl) 2 (lol)
Long range (|i - j| > 5)	49 (upl) 27 (lol)	49 (upl) 27 (lol)
**Violations**		
NOE distance violations > 0.5 Å	0	0
**Ramachandran Plot**		
Residues in most favored regions	52.4%	54.3%
Residues in additional allowed regions	82.9%	87.6%
**RMS deviation from mean structure (Å)**		
Backbone atoms (residue 1-14)	1.92	2.13
All heavy atoms (residue 1-14)	3.17	3.52

References

1. Jorgensen, W. L., et al. *J Chem Phys* **1983,** *79* (2), 926.
2. Tian, C., et al. *J Chem Theory Comput* **2020,** *16* (1), 528.
3. Huy, P. D., et al. *ACS Chem Neurosci* **2016,** *7* (10), 1348.
4. Pearlman, D. A., et al. *Comput Phys Commun* **1995,** *91* (1-3), 1.
5. Case, D. A., et al. *J Comput Chem* **2005,** *26* (16), 1668.
6. Hockney, R. W., et al. *J Comput Phys* **1974,** *14* (2), 148.
7. Ryckaert, J.-P., et al. *J Comput Phys* **1977,** *23* (3), 327.
8. Darden, T., et al. *J Chem Phys* **1993,** *98* (12), 10089.
9. Cohen, S. I. A., et al. *Proc Natl Acad Sci U S A* **2013,** *110* (24), 9758.
10. Knowles, T. P. J., et al. *Science* **2009,** *326* (5959), 1533.
11. Cohen, S. I. A., et al. *Nat. Struct. Mol. Biol.* **2015,** *22* (3), 207.
12. Roche, J., et al. *Biochemistry* **2016,** *55* (5), 762.
